# Liver X Receptor Activation with an Intranasal Polymer Therapeutic Prevents Cognitive Decline without Altering Lipid Levels

**DOI:** 10.1021/acsnano.0c09159

**Published:** 2021-03-05

**Authors:** María Eugenia Navas Guimaraes, Roi Lopez-Blanco, Juan Correa, Marcos Fernandez-Villamarin, María Beatriz Bistué, Pamela Martino-Adami, Laura Morelli, Vijay Kumar, Michael F. Wempe, A. C. Cuello, Eduardo Fernandez-Megia, Martin A. Bruno

**Affiliations:** † Instituto de Ciencias Biomédicas, Facultad de Ciencias Médicas, Universidad Católica de Cuyo, Av. José Ignacio de la Roza 1516, Rivadavia, 5400, San Juan, Argentina; ‡ National Council of Scientific and Technical Research (CONICET), Godoy Cruz 2290, C1425FQB Ciudad Autónoma de Buenos Aires Argentina; § Centro Singular de Investigación en Química Biolóxica e Materiais Moleculares (CIQUS) and Departamento de Química Orgánica, Universidade de Santiago de Compostela, Jenaro de la Fuente s/n, 15782 Santiago de Compostela, Spain; ∥ Laboratory of Brain Aging and Neurodegeneration, Fundación Instituto Leloir, IIBBA-CONICET, Av. Patricias Argentinas 435 C1405BWE, Ciudad Autónoma de Buenos Aires, Argentina; ⊥ School of Pharmacy, Department of Pharmaceutical Sciences, University of Colorado, Aurora, Colorado 80045 United States; # Department of Pharmacology and Therapeutics, McGill University, McIntyre Medical Building 3655 Prom. Sir-William-Osler, Montreal, Quebec H3G 1Y6, Canada

**Keywords:** Alzheimer’s disease, amyloid-beta, liver X receptor, DMHCA, dendrimer, polymeric micelle, drug delivery

## Abstract

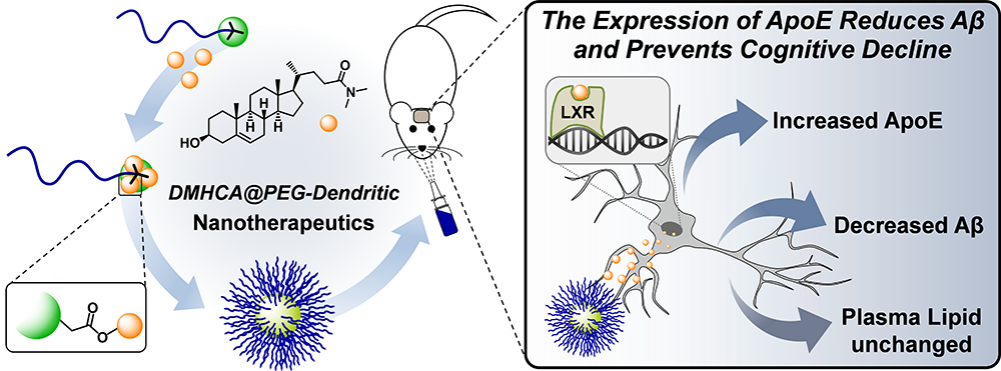

The progressive accumulation of amyloid-beta (Aβ) in specific areas of the brain is a common prelude to late-onset of Alzheimer’s disease (AD). Although activation of liver X receptors (LXR) with agonists decreases Aβ levels and ameliorates contextual memory deficit, concomitant hypercholesterolemia/hypertriglyceridemia limits their clinical application. DMHCA (*N*,*N*-dimethyl-3β-hydroxycholenamide) is an LXR partial agonist that, despite inducing the expression of apolipoprotein E (main responsible of Aβ drainage from the brain) without increasing cholesterol/triglyceride levels, shows nil activity *in vivo* because of a low solubility and inability to cross the blood brain barrier. Herein, we describe a polymer therapeutic for the delivery of DMHCA. The covalent incorporation of DMHCA into a PEG-dendritic scaffold via carboxylate esters produces an amphiphilic copolymer that efficiently self-assembles into nanometric micelles that exert a biological effect in primary cultures of the central nervous system (CNS) and experimental animals using the intranasal route. After CNS biodistribution and effective doses of DMHCA micelles were determined in nontransgenic mice, a transgenic AD-like mouse model of cerebral amyloidosis was treated with the micelles for 21 days. The benefits of the treatment included prevention of memory deterioration and a significant reduction of hippocampal Aβ oligomers without affecting plasma lipid levels. These results represent a proof of principle for further clinical developments of DMHCA delivery systems.

The progressive accumulation of amyloid-beta (Aβ) in specific areas of the brain is a common prelude to late-onset of Alzheimer’s disease (AD).^1^ The amyloid-cascade hypothesis has been for more than 25 years the central dogma for the development of AD.^2,3^ This hypothesis states than imbalance between the production and clearance of Aβ in the brain of affected individuals is responsible for neurodegeneration and dementia. Monomeric Aβ progressively aggregates into Aβ oligomers and finally into amyloid fibrils, found in AD plaques previously considered to be the cause of cognitive deficits. Since the amounts of Aβ fibrillar plaques do not correlate with cognitive decline,^4,5^ researchers have focused on the study of both soluble and membrane-associated Aβ oligomers to identify the Aβ form responsible for neurotoxicity. In this regard, strong evidence suggests that instead of monomer or Aβ fibrils, diffusible Aβ oligomers are largely suspected to be responsible for development and progression of cognitive deterioration characteristic of AD, causing direct injury to neurons, enhancing neuroinflammation, astrocytosis, gliosis, and eventually neuronal loss.^2,6,7^ As a result, the age-related impairment of Aβ homeostatic mechanisms has been postulated as a critical determinant of disease risk with even modest reductions in clearance of soluble Aβ resulting in elevated levels of Aβ oligomers and ultimately their progressive and chronic deposition within the brain. This process occurs while individuals are still cognitively normal. Thus, early intervention aimed to eliminating toxic Aβ oligomers in the brain offer a promising preventive therapeutic strategy not only to halt the development and progression of AD but also as a promising target for causal treatment of the disease.

The main contribution to Aβ drainage from the brain comes from apolipoprotein E (ApoE),^8,9^ the strongest genetic risk factor of AD,^10^ which modulates Aβ removal to the systemic circulation by its transport across the blood brain barrier (BBB).^11,12^ The interaction between ApoE and Aβ is conditioned by the correct lipidation of ApoE, which is induced by the ATP-binding cassette transporter A1 (ABCA1).^13,14^ When amyloid precursor protein (APP) Tg mice are crossed onto an ABCA1^–/–^ background, decreased ApoE lipidation and increased amyloid deposition are observed,^13,15,16^ whereas increasing ABCA1 favors ApoE lipidation and reduces amyloid deposition.^14^ Both ApoE and ABCA1 are modulated by cerebral expression of liver X receptors (LXRs) and mainly produced in the central nervous system (CNS) by astrocytes.^17^

Several studies have explored the potential utility of LXRs agonists in AD therapy.^13,14,17,18^*In vivo* studies using AD-like transgenic mouse models have revealed that LXRs agonists provoke an upregulation of ApoE and ABCA1 expression, a marked reduction in Aβ levels, enhanced brain cholesterol turnover, and reversed contextual memory deficits.^19−21^ However, a major concern with LXRs agonists is that they not only drive up transcription of ApoE and ABCA1 but also upregulate genes associated with fatty acid synthesis. As a result, the therapeutic application of LXRs agonists has been restricted due to undesirable side effects, promoting lipogenesis and triglyceride accretion through the activation of sterol-response element binding protein 1c (SREBP-1c) expression.^22^

DMHCA (*N*,*N*-dimethyl-3β-hydroxycholenamide, Figure 1) is a gene-selective LXR modulator that mediates potent transcriptional activation of ABCA1 and ApoE gene expression, while minimally affecting SREBP-1c.^22,23^ Thus, it represents an excellent therapeutic candidate for AD, circumventing the side effects of alternative LXRs agonists. Still, DMHCA’s very low solubility and inability to cross the BBB limits its application *in vivo*.^24^ To overcome these shortcomings and achieve enough delivery to target areas of the brain, herein we describe the covalent incorporation of DMHCA into a micellar polymer therapeutic, exploiting its unique hydroxyl group as chemical handle. Functionalization of a PEG-dendritic scaffold^25^ [PEG is poly(ethylene glycol), a linear, hydrophilic polymer, characterized by low toxicity and immunogenicity and widely used for biomedical applications]^26^ with DMHCA has afforded an amphiphilic copolymer with precise stoichiometry that efficiently self-assembles into nanometric micelles (Figure 1) for intranasal administration.

**Figure 1:**
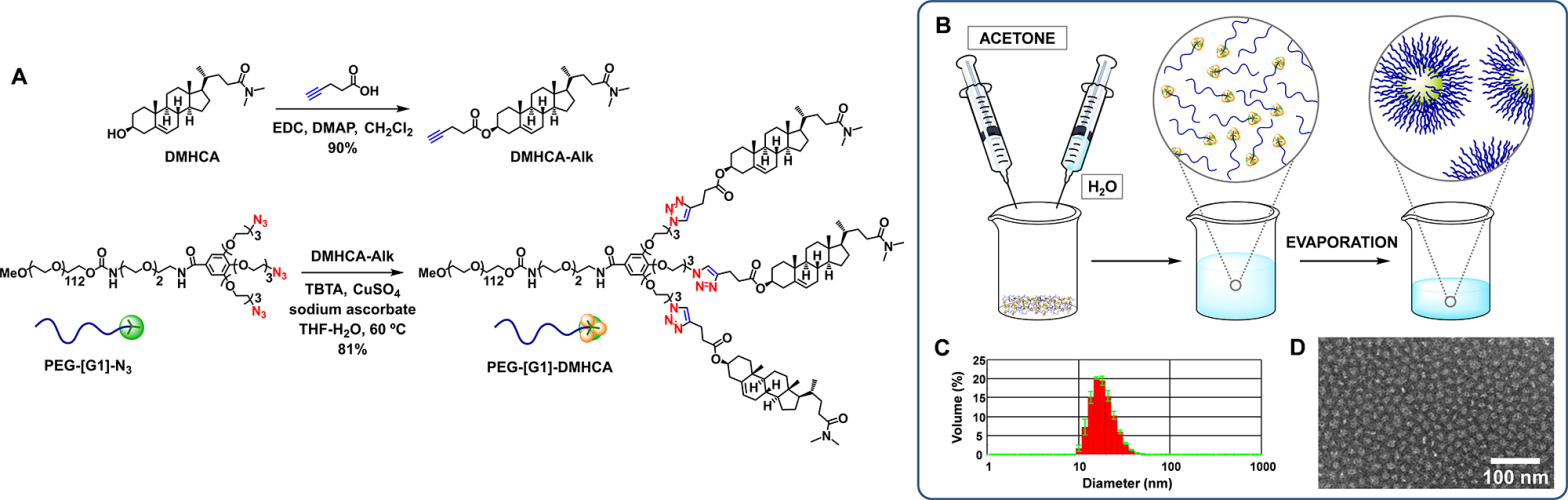
Synthesis of PEG-[G1]-DMHCA (A). Preparation of micelles (B). DLS histogram (C) and TEM image (D) of micelles.

## Results and Discussion

Figure 1 shows PEG-[G1]-DMHCA, a conjugate incorporating a PEG_5k_ chain, a GATG (gallic acid-triethylene glycol)^27−29^ dendritic block of first generation, and three pendant DMHCA molecules connected via carboxylate esters. These linkages were selected because of their known biodegradability *in vivo* by pH and the action of esterases and thus frequent use in the design of prodrugs.^30−32^ In addition, esters show a good compromise between biodegradability and synthetic manipulation. GATG dendrimers have been developed in our laboratory as a platform for biomedical applications.^27−29^ They are composed of a repeating unit incorporating a gallic acid core and hydrophilic triethylene glycol arms carrying terminal azides.^33^

The synthetic strategy toward PEG-[G1]-DMHCA has relied on an initial esterification of DMHCA with 4-pentynoic acid to afford DMHCA-Alk (EDC, DMAP, 90%), an alkynated derivative that was then connected via Cu(I)-catalyzed azide–alkyne cycloaddition (CuAAC)^34^ to PEG-[G1]-N_3_, a dendritic copolymer carrying three terminal azides, available in gram quantities in just one step^35^ (Figure 1). A proper selection of the reaction conditions and the presence of catalytic tris(benzyltriazolylmethyl) amine (TBTA) were determining factors to efficiently lead CuAAC to completion [CuSO_4_, ascorbate, TBTA, THF/H_2_O (4:1), 60 °C; see Table S1). PEG-[G1]-DMHCA was obtained in very good yield (81%) and chemically characterized with convincing evidence by ^1^H NMR (disappearance of the methylene protons adjacent to the azide at 3.40 ppm, appearance of new triazol protons at 7.51 ppm), ^13^C NMR (new triazol at 122.2 and 127.9 ppm), IR spectroscopy (loss of the intense azide band at 2100 cm^–1^), and MALDI-TOF MS (a series of 44 Da spaced peaks with *M*_p_ and *M*_w_ in agreement with expected values) as described in Supporting Information (SI). In addition, characteristic new signals in the ^1^H NMR [5.35 (alkene) and 3.00–2.93 ppm (*N*,*N*-dimethylamide)] and ^13^C NMR spectra [173.6 ppm (amide) and 139.5 ppm (alkene)] confirmed the incorporation of DMHCA.

Micellar assembles of PEG-[G1]-DMHCA were obtained by an evaporation method in acetone/H_2_O (1:1). Dynamic light scattering (DLS) measurements confirmed a mean diameter of about 22 ± 1 nm in agreement with transmission electron microscopy (TEM) images (Figures 1 and S2). These micelles containing a high 20% DMHCA drug loading were stable in solution for at least 1 week without variation in size or ester hydrolysis being observed (Figure S6). They could be freeze-dried and successfully resuspended in PBS (Figure S3), both relevant properties for storage and handling. Finally, to proceed with an *in vitro*/*in vivo* evaluation, a biotinylated version of the micelles was obtained from Biotin-PEG-[G1]-DMHCA, a copolymer carrying biotin at the distal end of the PEG block prepared following a similar strategy from BocHN-PEG-[G1]-N_3_ (a copolymer analogous to PEG-[G1]-N_3_ that incorporates a terminal protected amino group)^36^ (Scheme S1 and Figure S4).

**Figure 2:**
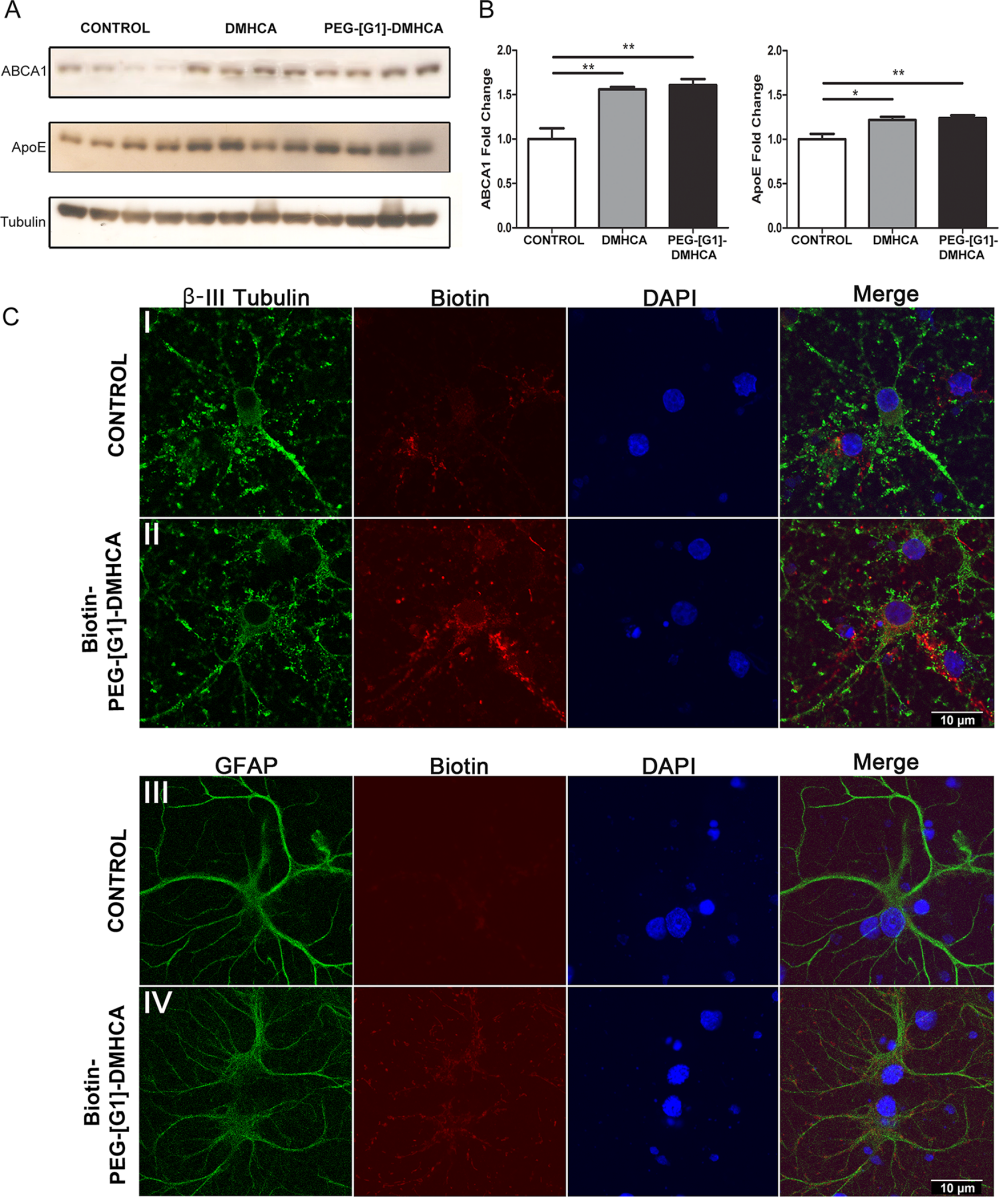
Biological effect of free DMHCA versus PEG-[G1]-DMHCA micelles versus PEG-[G1]-CO_2_H (control) over ABCA1 and ApoE levels tested on cerebral cortical cocultures treated for 24 h (A,B). Statics analysis were performed using GraphPad Prism 6. All probability values were two-tailed; a level of 5% was considered significant. Data are reported as the mean ± SEM. Confocal microscopy (C) of displaying neuronal (panel II; βIII-tubulin, green) and astrocytes (panel IV; GFAP, green) binding Biotin-PEG-[G1]-DMHCA (red) compared with controls (panels I and III, respectively). Nuclei stained in blue (DAPI). Scale bar, 10 μm.

To determine the therapeutic efficacy of PEG-[G1]-DMHCA, we compared the effect of DMHCA versus PEG-[G1]-DMHCA micelles on the ABCA1 and ApoE cell expression (PEG-[G1]-CO_2_H, the block copolymer resulting after hydrolysis of DMCHA was used as control). After 14 days *in vitro*, rat cortical neuronal and glial cocultured cells (800 000 cells per well) were treated for 24 h with 10 μM free or micellar DMHCA. Levels of ABCA1 and ApoE were resolved by Western blots (Figure 2A). As depicted in Figure 2B, there were statistically significant increases in both target proteins in free DMHCA treated cells versus control (*t* test *p* ≤ 0.01** and *p* ≤ 0.05*, respectively) and DMHCA micelles treated cells versus control (*t* test *p* ≤ 0.01** for both markers). The most striking finding is that both DMHCA and PEG-[G1]-DMHCA micelles upregulated target protein expression with respect to control with no statistical differences between them, confirming that PEG-[G1]-DMHCA micelles exert *in vitro* selective biological effects on ABCA1 and ApoE through LXRs activation.

Then, the target specificity of PEG-[G1]-DMHCA was evaluated by confocal imaging using the biotinylated version of the micelles. Neurons and astrocytes were incubated with Biotin-PEG-[G1]-DMHCA micelles (10 μM DMHCA) for 2 h. The distribution of Biotin-PEG-[G1]-DMHCA was examined, staining biotin with streptavidin-Alexa Fluor 594 (red) and nuclei with DAPI (blue). Neurons were marked with an anti-βIII-tubulin and a secondary antibody labeled with Alexa Fluor 488 (green). Biotin-PEG-[G1]-DMHCA displayed a strong signal and colocalized with neurons compared with control cells (Figure 2C, panels I and II). Similarly, astrocytes labeled with anti-GFAP (glial fibrillary acidic protein) and a secondary antibody-Alexa Fluor 488 (green) overlapped with Biotin-PEG-[G1]-DMHCA (red) (Figure 2C, panel III and control in panel IV). Overall, confocal imaging for primary cell culture displayed fluorescence intensity for both, neurons and astrocytes.

**Figure 3:**
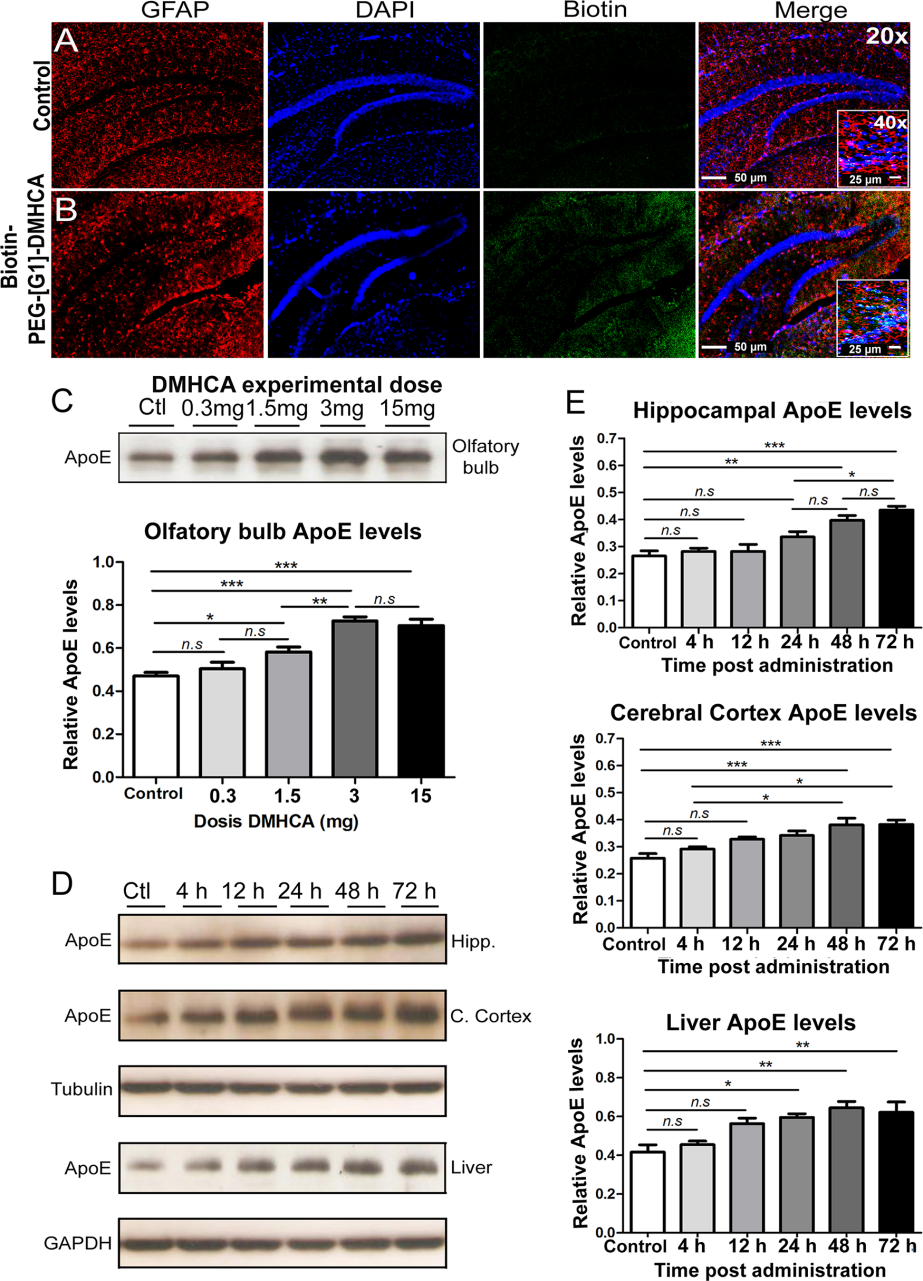
Biotin-PEG-[G1]-DMHCA intranasally administered reaches the hippocampus after 24 h. In the images, the colocalization of the used markers (red for GFAP/blue for DAPI, nuclei/green for Biotin) can be observed at 20× and 40× (scale bars 50 and 25 μm, respectively) (A,B). Effective intranasal dose analysis in mice: olfactory bulb levels of ApoE evaluated after 24 h by Western Blot (C). Relative levels of ApoE in homogenates of hippocampus, cerebral cortex, and liver; normalizing with values of βIII-tubulin for hippocampus and cerebral cortex, and GAPDH for liver (D,E). Statics analysis was performed using Graph-Pad Prism 6. All probability values were two-tailed; a level of 5% was considered significant. Data are reported as the mean ± SEM.

Having demonstrated the colocalization of PEG-[G1]-DMHCA with cells of the CNS *in vitro*, the *in vivo* administration of micelles was evaluated in mice. Drug delivery to the brain for the treatment of a wide variety of diseases has been traditionally hampered by the BBB. In recent years, the intranasal administration has come to light as an effective nose-to-brain passage that circumvents the BBB. The olfactory epithelium provides a direct pathway for the noninvasive, rapid, and comfortable delivery of therapeutic agents and drug delivery systems, including dendrimer nanoplatforms, to the CNS.^37−39^ Administration doses of 0.2, 5, and 10 mg DMHCA/kg body weight/day (equivalent to 1, 25, and 50 mg of DMHCA micelles/kg body weight/day) were tested by intranasal administration. Mice received 5 μL per nostril of a solution of Biotin-PEG-[G1]-DMHCA micelles and after 4, 12, and 24 h of administration, cerebral cortex and hippocampus were analyzed (Figure 3). At 24 h post intranasal administration, confocal images (20×) delivered an overlapped green signal from Biotin-PEG-[G1]-DMHCA (stained with streptavidin-Alexa Fluor 488) and an astrocytes red signal from GFAP (marked with anti-GFAP followed by a secondary antibody-Alexa Fluor 594) in treated mice versus controls both at 5 and 10 mg of DMHCA/kg body weight/day (Figure 3A,B). At higher magnification (40×), in some images of the hippocampus, Biotin-PEG-[G1]-DMHCA also overlapped with DAPI (blue), indicating accumulation in the nuclei, where LXRs are expressed (Figure 3B).

In previously reported studies of DMHCA, experimental doses in mice ranged from 8 to 80 mg/kg body weight/day for systemic or oral administration, without penetration through the BBB.^22,23,40^ We explored a range of four different doses of DMHCA, 0.3, 1.5, 3, and 15 mg/kg body weight/day, equivalent to 1.5, 7.5, 15, and 75 mg of PEG-[G1]-DMHCA/kg body weight/day. Animals were divided in five groups (*n* = 4 per group) and received a single intranasal dose with increasing concentrations of DMHCA micelles (10 μL total volume, 5 μL/nostril). After 24 h, mice were deeply anaesthetized and brains were rapidly removed. As depicted in Figure 3C, olfactory bulb levels of ApoE resolved by Western blots revealed that mice receiving 3 mg of DMHCA displayed increased relative ApoE levels compared to controls (*p* ≤ 0.001, ***) and the 0.3 mg (*p* ≤ 0.001, ***) and 1.5 mg (*p* ≤ 0.01, **) groups. However, no significant differences were observed between the 3 and 15 mg groups. Altogether, we concluded that 3 mg of DMHCA/kg body weight/day displayed an effective dose–response, triggering upregulation of ApoE levels. As expected, the intranasal treatment of free DMHCA at the same concentrations did not affect the levels of ApoE.

To check the effectiveness of the selected pharmacological dose of DMHCA micelles (3 mg DMHCA/kg body weight/day) at different times post intranasal administration, experimental mice were divided into six groups (*n* = 5 per group), as follows: group A (control), saline; group B, one dose administered at time 0 and sacrificed 4 h later; group C, one dose at time 0 and sacrificed after 12 h, group D, one dose at time 0 and sacrificed after 24 h; group E, two doses at time 0 and 24 h, sacrificed at 48 h post first administration; and group F, three doses at time 0, 24, and 48 h, sacrificed at 72 h post first administration. As shown in Figure 3D, relative ApoE levels were determined by Western blots in hippocampus, cerebral cortex, and liver. Quantification in Figure 3E shows a significant increase in ApoE levels at 48 h post initial administration in hippocampus and cerebral cortex. An increase in ApoE levels (*p* ≤ 0.05,*) at 24 h post initiation of administration is also observed in liver likely due to the high LXRs hepatic expression.

**Figure 4:**
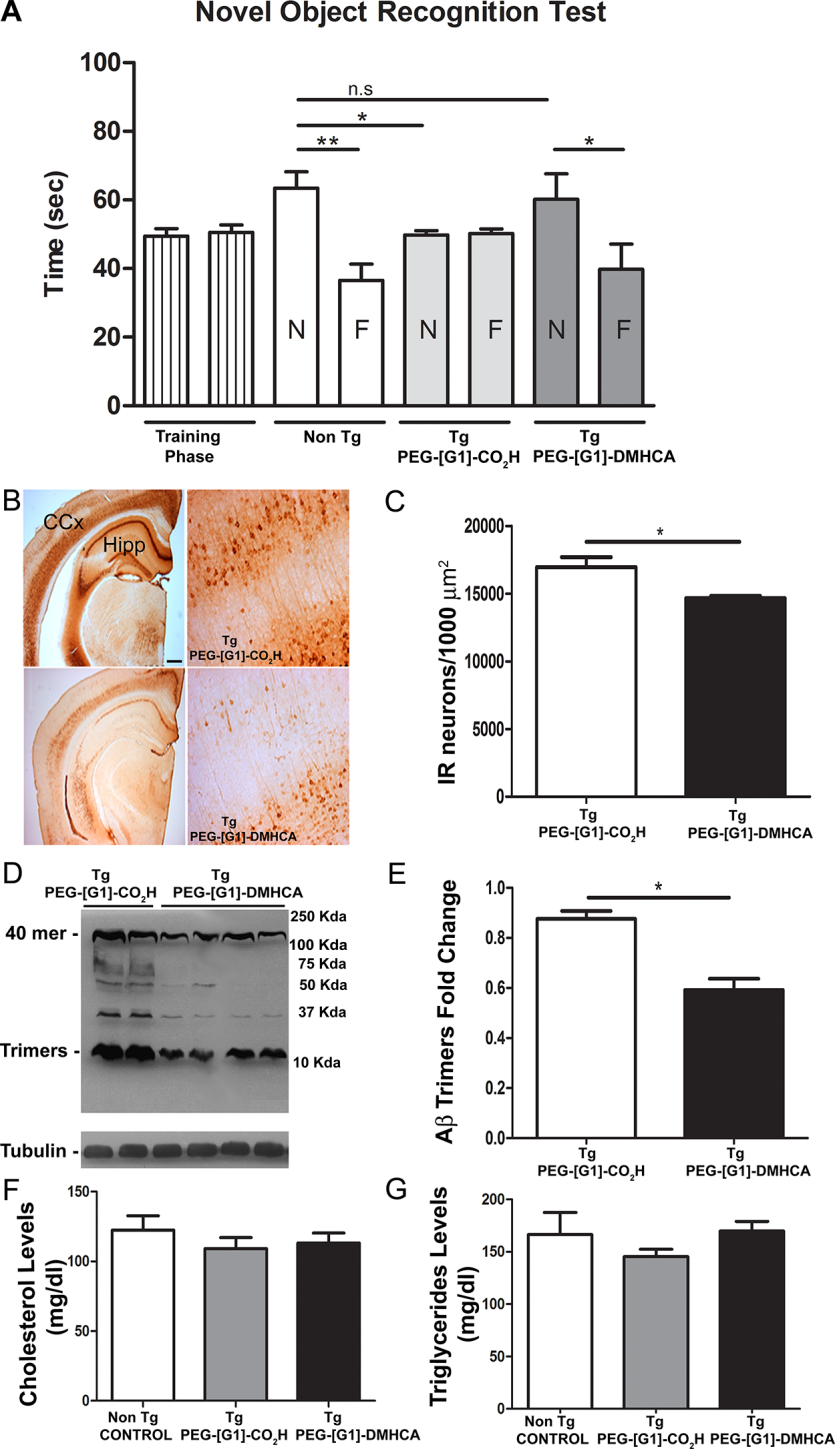
(A) Novel object recognition test: memory retention was tested 24 h after training. Data are mean ± SEM exploratory preferences during training (left columns) or test (white and gray columns; F, familiar; N, novel) trials (*n* = 8 per group). (B) Immunoreactive Aβ burden in the cerebral cortex (CCx) and hippocampus (Hipp) of PEG-[G1]-CO_2_H versus PEG-[G1]-DMHCA treated mice. (C) Positive immunoreactive neurons were quantified using the Image Pro Plus software. (D) Hippocampal homogenates were resolved by Western blots using the 6E10 antibody; a particular reduction of the 12 kDa band, referred as Aβ trimers (*p* ≤ 0.05,*, independent *t* test) was observed (E). Lipid plasma profile shows not statistically significant differences (independent *t* test) in the levels of cholesterol (F) and triglycerides (G) among groups. Statics analysis were performed using Graph-Pad Prism 6. All probability values were two-tailed; a level of 5% was considered significant. Data are reported as the mean ± SEM.

Next, we investigated the potential *in vivo* pharmacological effects of PEG-[G1]-DMHCA micelles on associated memory impairment and Aβ burden in our well-characterized AD-like amyloid pathology transgenic (Tg) mice.^41^ Three month old mice were divided into two groups. A control group received daily 10 μL (5 μL/nostril) intranasal administration of PEG-[G1]-CO_2_H (copolymer lacking DMHCA; 15 mg/kg body weight/day) for 21 consecutive days. The experimental group followed the same protocol and received 15 mg/kg body weight/day of DMHCA micelles (equivalent to 3 mg DMHCA/kg body weight/day). At the end of the treatment, we investigated whether chronic intranasal treatment with PEG-[G1]-DMHCA could prevent object recognition memory deficits characteristic of our Tg mice at this age [novel object recognition test (NOR)]. Gratifyingly, PEG-[G1]-DMHCA treated mice performed significantly better on the object recognition task than control mice (*p* < 0.05,*) and similar to nontransgenic age-matched littermates (Figure 4A). Figure 4B illustrates the immunoreactive Aβ burden in the right cerebral cortex and hippocampus of PEG-[G1]-CO_2_H versus PEG-[G1]-DMHCA treated mice. The study revealed a significant reduction (*p* ≤ 0.05,*) of Aβ positive neurons following PEG-[G1]-DMHCA treatment compared to control (Figure 4C). Left hippocampal homogenates were resolved by Western blots using the 6E10 antibody (Figure 4D). The analysis revealed several immunoreactive bands in Tg/PEG-[G1]-CO_2_H animals between 12 and 120 kDa, mainly oligomeric forms of Aβ not appearing in hippocampal Tg/PEG-[G1]-DMHCA homogenates. Clearly, the micellar DMHCA treatment results in the clearance of most of the hippocampal 6E10 immunoreactive bands with a particular reduction of the 12 kDa band, referred to as Aβ trimers (*p* ≤ 0.05,*; Figure 4E). Lastly, considering that significant efforts are currently directed toward developing LXRs ligands that lack an undesired upregulation of hepatic lipogenesis, the lipid plasma profile was studied, revealing no statistically significant differences in the plasma levels of cholesterol and triglycerides among groups (Figure 4F,G).

Finally, the potential cytotoxicity of the intranasal treatment with DMHCA micelles was evaluated in mice using a caspase 3/7 assay. Apoptosis is the process of programmed cell death that occurs in all living organisms. Detecting apoptosis is key to determine mechanisms of cell toxicity. In mammalian cells, apoptosis is accompanied by an increased production of caspases, enzymes responsible for the activation of signaling pathways and the proteolytic dismantling of key processes ultimately leading to cell death. To study the potential cytotoxic effects *in vivo* of PEG-[G1]-DMHCA micelles, we have investigated the specific activation of two effector caspases, caspase-3 and 7, which are downstream of the initiator events of the apoptotic cascade (see the SI). The analysis of homogenates of the olfactory bulb, hippocampus, cerebral cortex and liver of mice treated with PEG-[G1]-DMHCA micelles at different time-points (up to 72 h) indicates that the treatment does not mediate *in vivo* cell toxicity through an apoptotic mechanism at any of the experimental doses (Figure S7). The cytotoxic effect of the long-term PEG-[G1]-DMHCA treatment was also studied in 21 days-treated transgenic mice (Figure S8). Our studies do not reveal any statistical differences in caspase 3/7 activity between tissues [brain (olfactory bulb, hippocampus, and cerebral cortex), liver, lungs, and heart] of non Tg and Tg mice treated with PEG-[G1]-CO_2_H (control) or PEG-[G1]-DMHCA micelles. Overall, our results indicate that the long-term intranasal treatment of PEG-[G1]-DMHCA micelles at the experimental dose (3 mg/kg body weight/day) does not trigger caspase-related cell toxicity mechanisms in our mice model.

## Conclusions

The age-related impairment of Aβ homeostatic mechanisms has been postulated as a critical determinant of disease risk in AD with even modest reductions in the clearance of soluble Aβ resulting in elevated levels of toxic oligomers, and ultimately their progressive and chronic deposition within the brain. DMHCA represents an LXR partial agonist that despite inducing the expression of ApoE (mainly responsible of Aβ drainage from the brain) shows nil activity *in vivo* because of low solubility/inability to cross the BBB. Our DMHCA polymer therapeutic approach of intranasally administered dendritic micelles at very early stages of the pathology effectively prevents cognitive deficits assessed by the NOR test and reduces Aβ deposition without undesirable side effects, leaving the plasma levels of cholesterol and triglycerides unaffected. We believe these studies render suitable proof of principle for further successful clinical applications of DMHCA delivery systems.

## Experimental Section

### Synthesis of DMHCA-Alk

DMHCA (100 mg, 0.25 mmol), DMAP (6.1 mg, 49.8 μmol), and EDC·HCl (62 mg, 0.32 mmol) were added to a solution of 4-pentynoic acid (29 mg, 0.30 mmol) in CH_2_Cl_2_ (0.5 mL) under Ar. After 20 h of stirring at room temperature, the solvent was evaporated and the mixture was diluted with CH_2_Cl_2_ (15 mL) and washed with 0.5 M HCl (2 × 10 mL) and brine (15 mL). The organic layer was dried (MgSO_4_) and concentrated to give a crude product that was purified by automated MPLC (gradient from hexane to EtOAc, silica, 15 min) to afford DMHCA-Alk (109 mg, 90%) as a white crystalline solid. ^1^H NMR (400 MHz, CDCl_3_) δ: 5.37 (d, *J* = 4.6 Hz, 1H), 4.71–4.56 (m, 1H), 3.00 (s, 3H), 2.93 (s, 3H), 2.57–2.44 (m, 4H), 2.41–2.14 (m, 4H), 2.05–0.88 (m, 28H), 0.68 (s, 3H). ^13^C NMR (100 MHz, CDCl_3_) δ: 173.8, 171.3, 139.7, 122.8, 82.7, 74.5, 69.1, 56.8, 56.0, 50.1, 42.5, 39.8, 38.2, 37.5, 37.1, 36.7, 35.7, 35.5, 33.8, 32.0, 31.4, 30.5, 28.3, 27.9, 24.4, 21.2, 19.4, 18.7, 14.6, 12.0. IR (ATR): 3321, 2933, 2851, 1733, 1625 cm^–1^. ESI-MS (*m*/*z*): 482.3632. Calcd for [M + H]^+^, C_31_H_47_NO_3_: 482.3634.

### Synthesis of PEG-[G1]-DMHCA

DMHCA-Alk (49 mg, 0.10 mmol) was added to a solution of PEG-[G1]-N_3_ (100 mg, 17 μmol) in a mixture of THF (0.41 mL) and H_2_O (26 μL). Then, TBTA (2.7 mg, 5.10 μmol), CuSO_4_ (12.8 μL, 2.55 μmol, 0.2 M, 5 mol % per azide), and sodium ascorbate (64.0 μL, 12.80 μmol, 0.2 M, 25 mol % per azide) were added. After 12 h of stirring at 60 °C, a second portion of sodium ascorbate (64.0 μL, 12.80 μmol, 0.2 M, 25 mol % per azide) was added. After additional 24 h of stirring at 60 °C, the reaction mixture was partitioned between CH_2_Cl_2_ (15 mL) and 0.1 M EDTA pH 7 (15 mL). The organic layer was washed again with 0.1 M EDTA pH 7 (2 × 15 mL) and brine (15 mL). Then, it was dried (MgSO_4_), evaporated, and purified by precipitation (CH_2_Cl_2_/Et_2_O) to afford PEG-[G1]-DMHCA (100 mg, 81%) as a white solid. ^1^H NMR (750 MHz, CDCl_3_) δ: 7.51 (s, 3H), 7.09 (s, 2H), 5.35 (d, *J* = 5.1 Hz, 3H), 4.63–4.54 (m, 3H), 4.52–4.42 (m, 6H), 4.26–4.07 (m, 8H), 3.90–3.50 (m, ∼484H), 3.40–3.31 (m, 7H), 3.04–2.96 (m, 15H), 2.93 (s, 9H), 2.72–2.64 (m, 6H), 2.35 (ddd, *J* = 15.6, 11.0, 5.1 Hz, 3H), 2.28 (d, *J* = 8.2 Hz, 6H), 2.20 (ddd, *J* = 14.8, 10.7, 5.6 Hz, 3H), 2.02–1.92 (m, 6H), 1.91–1.75 (m, 12H), 1.61–1.40 (m, 20H), 1.36–0.90 (m, 43H), 0.67 (s, 9H). ^13^C NMR (100 MHz, CDCl_3_) δ: 173.6, 172.1, 166.9, 156.4, 152.3, 146.2, 139.5, 129.0, 127.9, 122.6, 122.2, 107.0, 74.1, 72.3, 71.9, 70.8, 70.2, 69.5, 68.8, 63.9, 59.0, 56.6, 55.9, 50.1, 49.9, 42.3, 39.6, 38.0, 37.3, 36.9, 36.5, 35.6, 35.4, 34.0, 31.8, 31.2, 30.3, 28.1, 27.7, 24.2, 21.0, 19.3, 18.5, 11.8. IR (ATR): 3523, 2868, 1730, 1640, 1104 cm^–1^. MALDI-TOF MS (HABA, linear mode, *m*/*z*): Calcd, *M*_p_ 7251 ([M + H]^+^), *M*_n_ 7278; Found, *M*_p_ 7258 ([M + H]^+^), *M*_n_ 7243, *M*_w_ 7272.

### Preparation of DMHCA Micelles

PEG-[G1]-DMHCA was dissolved in a mixture of acetone/H_2_O (1:1, 0.5 mg/mL) and stirred at room temperature for 48 h until acetone was completely evaporated. The resulting micelles (1 mg/mL) were freeze-dried. Biotin-PEG-[G1]-DMHCA micelles with a 10% biotin loading were prepared following the same procedure as above from a mixture of PEG-[G1]-DMHCA and Biotin-PEG-[G1]-DMHCA in a molar ratio 9:1. DLS histograms and correlation functions of the micelles as prepared and after resuspension in 10 mM PB, pH 7.4, 150 mM NaCl (1 mg/mL) are shown in Figure S2–S4.

^1^H NMR analysis of the micelles revealed at a glance that there is a core–corona structure (Figure S5). Only resonances for the flexible PEG chains at the hydrophilic corona are visible, whereas nuclei from DMHCA and the dendritic block are absent from the spectrum as a result of their restricted mobility at the compact core. Interestingly, no ester hydrolysis is observed during the preparation/storage of the micelles as revealed by the ^1^H NMR of a lyophilized sample of micelles after being redissolved in CDCl_3_ (Figure S6).

### Primary Neuronal/Glial Cultures

Primary cortical cells (neurons cocultured with glia) were obtained from embryonic rats using a standard procedure. Briefly, 8–10 embryos E15–16 were extracted from the uterus of pregnant Wistar rats and cerebral cortices were isolated in HBSS buffer (Thermo Scientific). The tissue was incubated with 2 mL of 0.25% trypsin-EDTA (Thermo Scientific) for 15 min at 37 °C and then washed twice with DMEM/F12–10% FBS. The medium was replaced by Neurobasal (Thermo Scientific) and cortices were homogenized by pipetting up and down. After that, cells were incubated for 10 min at room temperature and then centrifuged at 200×*g* for 5 min. The medium was discarded and replaced with 2 mL of neuronal medium (Neurobasal, 2 mM l-glutamine, 2% B27 (Thermo Scientific), 100 U/mL penicillin, 100 μg/mL streptomycin). Cells were resuspended, and viability was assessed with Trypan blue dye. Then, cells were plated in previously poly-l-lysine coated 12 mm-diameter coverslips for immunofluorescence experiments (150 000 cells per coverslip) or 6-multiwell plates for Western blot experiments (800 000 cells per well) and maintained in a 37 °C humidified incubator with 5% CO_2_ until DIV (days *in vitro*) 14.

### *In Vitro* Biological Effects

The primary cell culture was plated at 800 000 cells per well in 6-well plates. Immediately, PEG-[G1]-CO_2_H (control), free DMHCA, or PEG-[G1]-DMHCA micelles were added to the conditioned medium of DIV14 cells to a final concentration of 10 μM DMHCA. Cultures were then incubated in a 37 °C humidified incubator with 5% CO_2_ for 24 h. After incubation, the conditioned medium was aspirated and cells (neurons and glia) were washed in cold PBS and lysed in RIPA buffer containing protease inhibitors (SigmaFast protease inhibitor, St. Louis, MO).

### Cell Uptake Experiment and Fluorescence Imaging *In Vitro*

Biotin-PEG-[G1]-DMHCA micelles were added to the conditioned medium of DIV14 cells to a final concentration of 10 μM of DMHCA, and cultures were then incubated in a 37 °C humidified incubator with 5% CO_2_ for 2 h. Then, the conditioned medium was aspirated, and cultures were washed with cold PBS and fixed in 4% PFA. Cells were incubated with primary antibodies anti-βIII-tubulin (1:1000, mouse, Promega) or antiglial fibrillary acidic protein (GFAP 1:1000, rabbit, Dako) overnight at 4 °C, followed by incubation with secondary antibodies labeled with Alexa Fluor 488 (green) or streptavidin-Alexa Fluor 594 (red). Coverslips were mounted with Mounting Medium with DAPI (blue) and images were obtained with a confocal Zeiss LSM 510 Meta microscope with a 40× oil-immersion lens and analyzed with LSM5 image browser software.

### Confocal Fluorescence Imaging

Mice received a single intranasal dose of Biotin-PEG-[G1]-DMHCA micelles (0.6 mg in 10 μL PBS), and 24 h later their brains were perfused-fixed. Then, 30 μm thick sections containing the hippocampus and cerebral cortex regions were prepared, and double immunostaining was performed to identify astrocytes and Biotin-PEG-[G1]-DMHCA. DAPI was added to stain nuclei (blue). Astrocytes were identified with rabbit antiglial fibrillary acidic protein (GFAP 1:1000, rabbit, Dako) followed by incubation with secondary antibodies labeled with Alexa Fluor 594 (red). Biotin-PEG-[G1]-DMHCA was identified using streptavidin-Alexa Fluor 488 (green). In all cases, sections were preincubated, blocked with the corresponding normal serum secondary antibody and coverslips were mounted with Gelvatol. The colocalization of Biotin-PEG-[G1]-DMHCA and astrocytes marker images were obtained with a confocal Zeiss LSM 510 Meta microscope with a 40× oil-immersion lens and analyzed with LSM5 image browser software.

### Animals, Intranasal Delivery Dose–Response, and Treatment Experiments

Three-month-old mice (*n* = 4–5/condition) were weighed and assigned to treatment groups. Mice received daily intranasal delivery of control (PEG-[G1]-CO_2_H or saline) or PEG-[G1]-DMHCA micelles in PBS. The intranasal delivery was performed according to the protocol described by Hanson et al.^42^ First, mice were subjected to simulated delivery for 1 week before treatments to reduce the stress due to the procedure.

For intranasal delivery, mice were hand-restrained, placed in a supine position, and given two 5 μL drops of PEG-[G1]-DMHCA micelles, or a control solution, into both nostrils consecutively. Mice were given an extra 5 μL treatment drop if the subject forcibly ejected or sneezed out the solution. Mice were held supine for 5–10 s after delivery to ensure that all fluid was inhaled. These volumes have shown to deliver drugs mostly to the brain without passage to the pulmonary regions. For dose–response experiments, mice received a range of 0.01 to 0.5 mg DMHCA per animal contained into micelles (equivalent to 0.3–15 mg DMHCA/kg body weight/day).

For treatment experiments, McGill-Thy1-APP transgenic (Tg) mice were three months old when they started the treatment and were sacrificed 21 days after. Young, preplaque three month old Tg mice received 15 mg/kg body weight/day of PEG-[G1]-CO_2_H (Tg-control) or 15 mg/kg body weight/day of PEG-[G1]-DMHCA micelles by intranasal administration for a 3 week period (non Tg control, *n* = 5 received intranasal administration of PBS; Tg/PEG-[G1]-CO_2_H, *n* = 5; Tg/PEG-[G1]-DMHCA micelles, *n* = 5). The animals were housed in groups of up to four in individually ventilated cages under standard conditions (22 °C, 12 h light–dark cycle) receiving food and water ad libitum. All procedures were approved by the Animal Care Committee of the Catholic University of Cuyo, Argentine and followed the guidelines of the Argentinean Council on Animal Care.

### Perfusion and Tissue Preparation Technique

Experimental mice were deeply anesthetized with equithesin (pentobarbital-based, 2.5 mL/kg, i.p.) and perfused through the heart with ice-cold saline solution (pH 7.4) for 1 min. The brains were then quickly removed and divided into right and left hemispheres on ice. The cortex, hippocampus, and olfactory bulb were dissected from the left hemisphere, snap-frozen in dry ice, and stored at −80 °C for biochemical analysis. The same treatment was applied to the liver. The right hemisphere was fixed in 4% paraformaldehyde (PFA) in 0.1 M phosphate buffer (PBS, pH 7.4) for 24 h at 4 °C. The tissue was then cut into 30 μm thick sections with a freezing sledge microtome (SM 2000R, Leica) at −20 °C and free-floating sections were collected in PBS and processed for immunofluorescence.

### Plasma Lipid Parameters

Blood was withdrawn intracardially and EDTA-plasma was prepared within 20 min. Plasma TG (Wiener lab, Argentine) and total cholesterol (Wiener lab, Argentine) concentrations were measured enzymatically.

### Novel Object Recognition Test

The NOR (Novel Object Recognition) was performed according to established protocols.^43^ Briefly, three month old mice, Tg and non Tg subjected to treatment, were habituated first to an empty open field box of 50 cm × 50 cm × 30 cm (for 10 min). After 24 h of the habituation session, mice were exposed to two identical nontoxic “familiar” objects (culture bottles filled with sand, T25). Between each test, the open field was cleaned with 70% ethanol to eliminate olfactory signals. After a retention interval of 4 h, animals were again exposed to the field where two objects were located, one familiar and one novel (insert cube, red). The definition of exploring that was used consists of detecting that the mouse was sniffing, climbing or touching the object or was at a distance of at least 3 cm from the object, while facing it or facing it. Each session lasted 10 min, and during this time mice were allowed to interact freely with the objects and the amount of exploration time of each object was recorded with the HVS Image software. The objects were randomized and counterbalanced through the animals. Animals that spent less than 7 s exploring objects during the 10 min training session were excluded from the analysis.

## Supporting Information Available

Materials, instrumentation, synthesis of DMHCA, Table S1, characterization of DMHCA-Alk and PEG-[G1]-DMHCA, synthesis and characterization of Biotin-PEG-[G1]-DMHCA, DLS and NMR characterization of PEG-[G1]-DMHCA micelles, Western blotting, immunohistochemistry and data analysis (PDF)
