# Supplementary material for: Liver X Receptor Activation with an Intranasal Polymer Therapeutic Prevents Cognitive Decline without Altering Lipid Levels
Source: ACS Nano. 2021 Mar 5;15(3):4678–87. doi: 10.1021/acsnano.0c09159 (PMC8488954; doi:10.1021/acsnano.0c09159)
Supplement: Supplementary file 1 — nn0c09159_si_001.pdf [file nn0c09159_si_001.pdf]

Supporting Information

Liver X Receptor Activation with an Intranasal Polymer Therapeutic Prevents Cognitive Decline without Altering Lipid Levels

María Eugenia Navas Guimaraes,[†,‡] *Roi Lopez-Blanco,*[§] *Juan Correa,*[§] *Marcos Fernandez-Villamarin,*[§] *María Beatriz Bistué,*[†] *Pamela Martino-Adami,*[#] *Laura Morelli,*[#] *Vijay Kumar,*[ǂ] *Michael F. Wempe,*[ǂ] *A. C. Cuello,*[⁋] *Eduardo Fernandez-Megia,**[,§] *and Martin A. Bruno**[,†,‡]

[†]Instituto de Ciencias Biomédicas, Facultad de Ciencias Médicas, Universidad Católica de Cuyo, Av. José Ignacio de la Roza 1516, Rivadavia, 5400, San Juan, Argentina.

[‡]National Council of Scientific and Technical Research (CONICET), Godoy Cruz 2290, C1425FQB Ciudad Autónoma de Buenos Aires, Argentina.

[§]Centro Singular de Investigación en Química Biolóxica e Materiais Moleculares (CIQUS) and Departamento de Química Orgánica, Universidade de Santiago de Compostela, Jenaro de la Fuente s/n, 15782 Santiago de Compostela, Spain.

[#]Laboratory of Brain Aging and Neurodegeneration, Fundación Instituto Leloir, IIBBA-CONICET, Av. Patricias Argentinas 435, C1405BWE Ciudad Autónoma de Buenos Aires, Argentina.

[ǂ]School of Pharmacy, Department of Pharmaceutical Sciences, University of Colorado, Aurora, Colorado, 80045 USA.

[⁋]Department of Pharmacology and Therapeutics, McGill University, McIntyre Medical Building 3655 Prom. Sir-William-Osler, Montreal, QC H3G 1Y6, Canada.

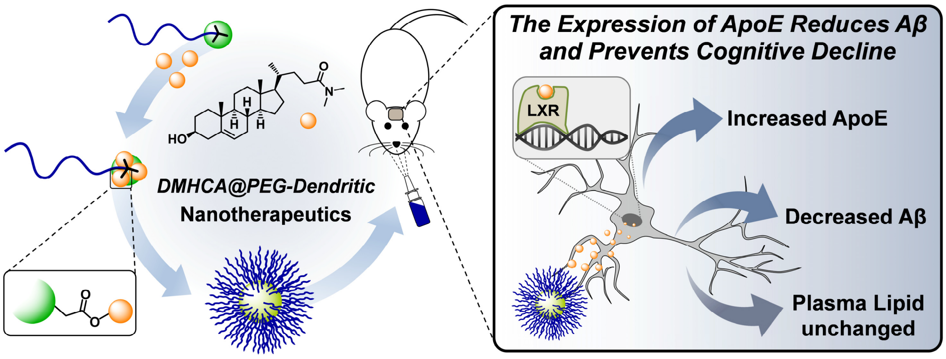

Table of Contents

1. Materials S3
2. Instrumentation S3
3. Synthesis of DMHCA S6
4. Table S1 .... S6
5. Characterization of DMHCA-Alk and PEG-[G1]-DMHCA S7
6. Synthesis and Characterization of Biotin-PEG-[G1]-DMHCA S11
7. Assignment of Signals in IR Spectra S16
8. DLS of PEG-[G1]-DMHCA and Biotin-PEG-[G1]-DMHCA Micelles S17
9. ^{1}H NMR Spectrum of PEG-[G1]-DMHCA Micelles S19
10. Integrity of the Ester Bond in PEG-[G1]-DMHCA Micelles by ^{1}H NMR S20
11. Western Blotting S22
12. Immunohistochemistry S22
13. *In Vivo* Cytotoxicity S23
14. Data Analysis S25
15. References S26

1. Materials

All chemicals were purchased from Sigma-Aldrich or Fluka unless otherwise noted. All solvents were HPLC grade, purchased from Scharlab, Sigma-Aldrich or Acros Organics. Et_3N was dried under 4Å molecular sieves. DMF and CH_2Cl_2 were dried using a SPS800 solvent purification system from MBRAUN. H_2O of Milli-Q grade was obtained from a Millipore water purification system. PEG-[G1]-N_3,[1,2] BocHN-PEG-[G1]-N_3,[3] and control treatment PEG-[G1]-CO_2H[4,5] with PEG_{5k} were prepared following previously reported procedures. DMEM media, antibiotic-antimycotic (AA), fetal bovine serum (FBS), trypsin/EDTA solution, and methanol-free formaldehyde (10%) were purchased from Life Technologies. Poly-L-lysine-treated glass coverslips were purchased from Becton Dickinson (Franklin Lakes, NJ). Rabbit anti-ApoE antibody, Rabbit anti-ABCA1 and Mounting Medium with DAPI were purchased from Abcam and goat anti-rabbit Fc antibody from Biorad. Anti-biotin (Jackson Immuno Research) and Anti-GFAP from Dako.

2. Instrumentation

Column Chromatography. Automated column chromatography was performed on a MPLC Teledyne ISCO CombiFlash RF 200 psi with RediSep Rf normal-phase 12 g silica columns.

Ultrafiltration. Purifications by ultrafiltration were performed on Millipore Amicon stirred cells using Amicon YM3 regenerated cellulose membranes (MWCO 3 kDa) under 5 psi N_2 pressure.

NMR Spectroscopy. NMR spectra were recorded on Varian Inova 400 MHz, Bruker DRX 500 MHz and Bruker NEO 750 MHz spectrometers. Chemical shifts are reported in ppm (δ units) downfield from the HOD solvent peak (D_2O) or internal tetramethylsilane ($CDCl_3$). All spectra were processed using Mestre Nova 9.0.0 software (Mestrelab Research).

Infrared Spectroscopy. FT-IR spectra were recorded on a Perkin-Elmer Spectrum Two equipped with a UATR accessory.

Mass Spectrometry. ESI-MS mass spectra were collected on a Bruker Microtof spectrometer. MALDI analysis were performed in a 4800 MALDI-TOF/TOF analyzer (Applied Biosystems, Foster City, CA, USA). PEG-[G1]-DMHCA was dissolved in 50 μL of $CHCl_3$. Then, different dilutions (sample: matrix) were made: 1 μL of polymer solution was mixed with 20 μL (1:20 dilution) or 40 μL (1:40 dilution) of a 2-(4-hydroxyphenylazo)benzoic acid (HABA) solution (0.05 M in dioxane) and analyzed by MALDI-TOF. 1 μL aliquots were deposited using the dried-droplet method onto a 384 Opti-TOF MALDI plate (Applied Biosystems, Foster City, CA, USA). MS spectra were acquired in linear mode (20 kV source) with a Nd:YAG, (355 nm) laser, and averaging 1000 laser shots.

Dynamic Light Scattering (DLS). DLS measurements were carried out on a Malvern Nano ZS (Malvern Instruments, U.K.) operating at 633 nm with a 173º scattering angle, at 25 ºC. Hydrodynamic diameters of micelles (1 mg/mL) were measured at 25 ºC in H_2O or 10 mM PB, pH 7.4, 150 mM NaCl. Mean diameters were obtained from the volume particle size distribution provided by Malvern Zetasizer Software.

Transmission Electron Microscopy (TEM). TEM measurements were performed on a JEOL JEM-1011 operated at 100 kV electron microscope equipped with a camera

MegaView G2. A drop of a solution of micelles (0.1 mg/mL) was settled on a PELCO® TEM carbon type-B film copper grid (Ted Pella, Inc.) and allowed to dry at room temperature for 12 h. Negative staining was performed by using a droplet of 2% uranyl acetate following standard procedures. The size of the micelles was determined with ImageJ software (version 1.51j8) measuring the line intensity profile across the assemble. An average diameter of 17±2 nm was obtained by measuring the size of 25 micelles.

3. Synthesis of DMHCA

CH_2Cl_2 (50 mL) was purged at 0 °C with dimethylamine (gas) for 5 min with slow flow rate. Then, 3β-hydroxy-Δ^5-cholenic acid (500 mg, 1.33 mmol), DCC (329.3 mg, 1.60 mmol) and DMAP (16.3 mg, 0.13 mmol) were added under N_2. The temperature was raised to room temperature and the reaction mixture was stirred for 48 h. Then, the solvent was evaporated and EtOAc (100 mL) was added. The resulting mixture was filtered and sequentially washed with sat $NaHCO_3$ (50 mL), sat NH_4Cl (50 mL) and brine (50 mL), dried (Na_2SO_4) and evaporated. The residue was purified by column chromatography (gradient from hexane/EtOAc 1:3 to 1:4, silica) to give DMHCA (400 mg, 75%) with identical NMR data to that previously reported.[6]

4. Table S1

Table S1. Optimization of CuAAC coupling conditions (6 equiv DMHCA-Alk, 5 mol% $CuSO_4$ per azide, THF:H_2O, 4:1).

TBTA (mol% per N_3)	[N_3] (M)	T (ºC)	t (h)	Conversion (%)*
-	0.05	rt**	24	0
0.10	0.05	rt**	168	43
0.10	0.05	60	120	93
0.10	0.10	60	36	100

* Conversion by ^{1}H NMR.
** Room temperature.

5. Characterization of DMHCA-Alk and PEG-[G1]-DMHCA

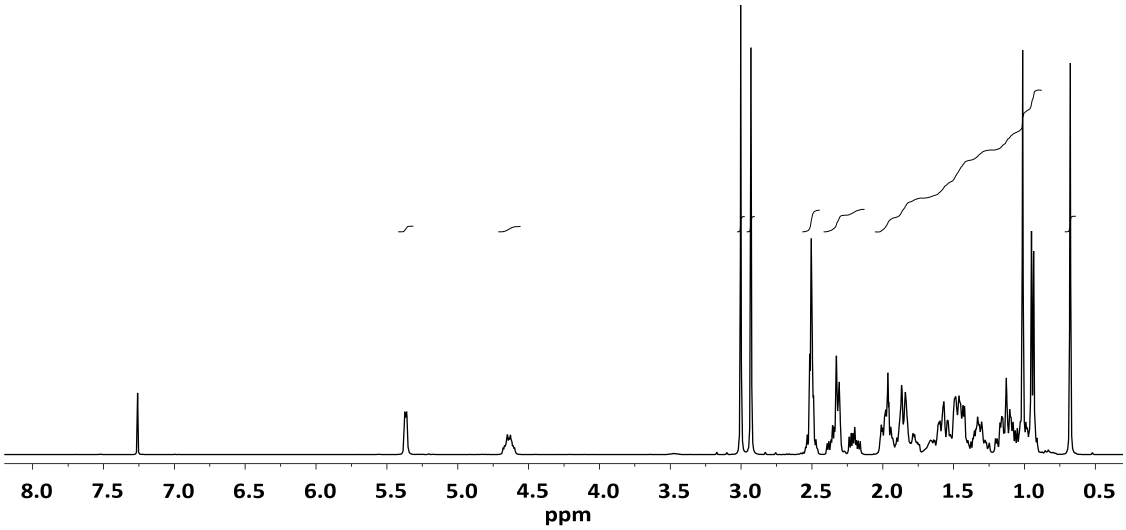

^{1}H NMR spectrum ($CDCl_3$, 400 MHz) of DMHCA-Alk.

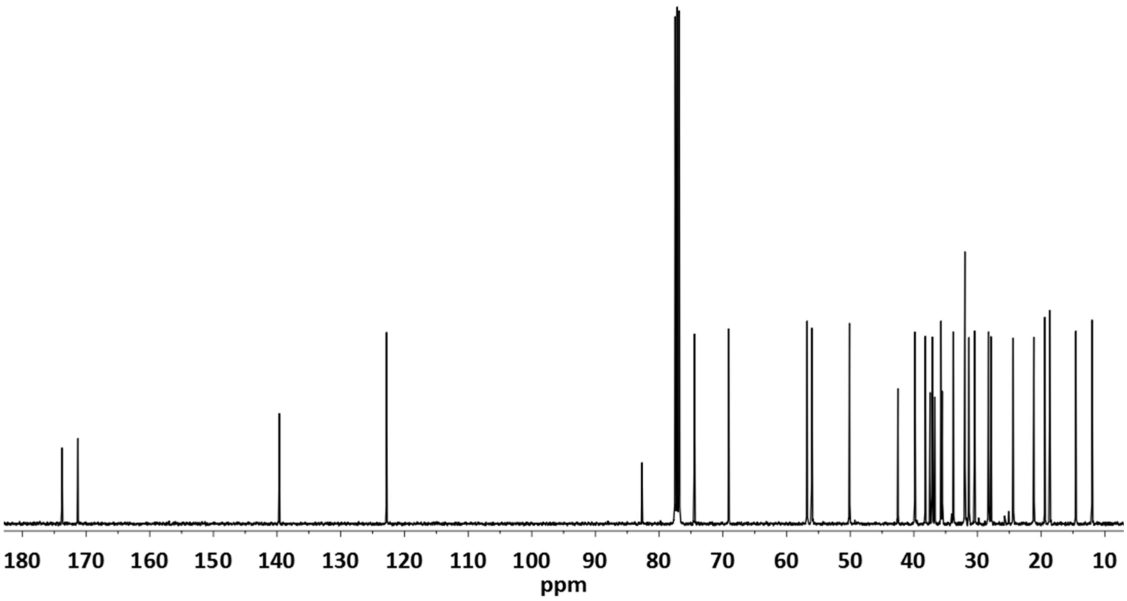

^{13}C NMR spectrum ($CDCl_3$, 100 MHz) of DMHCA-Alk.

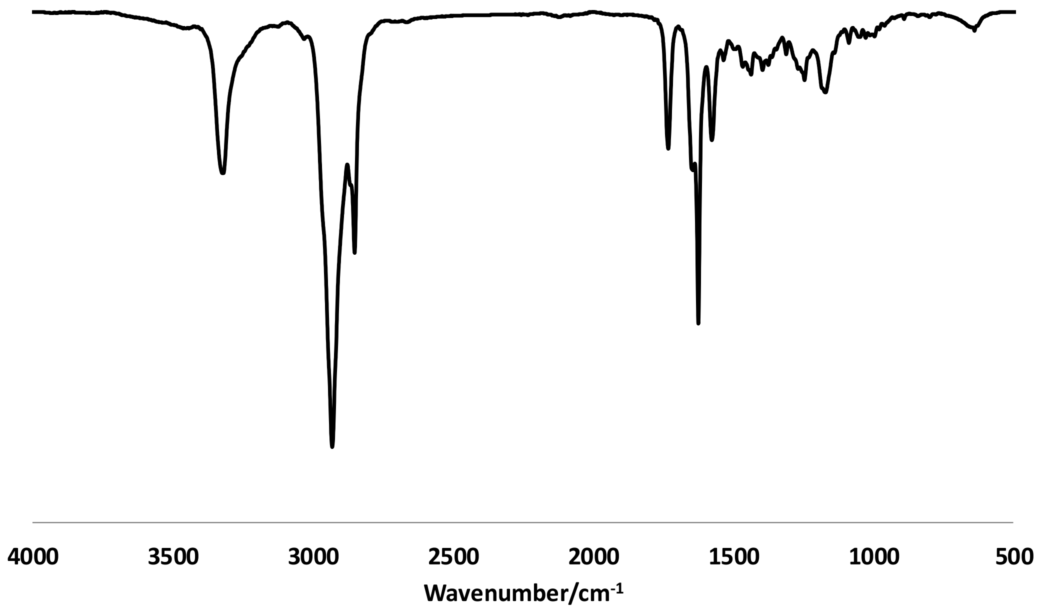

IR spectrum of DMHCA-Alk.

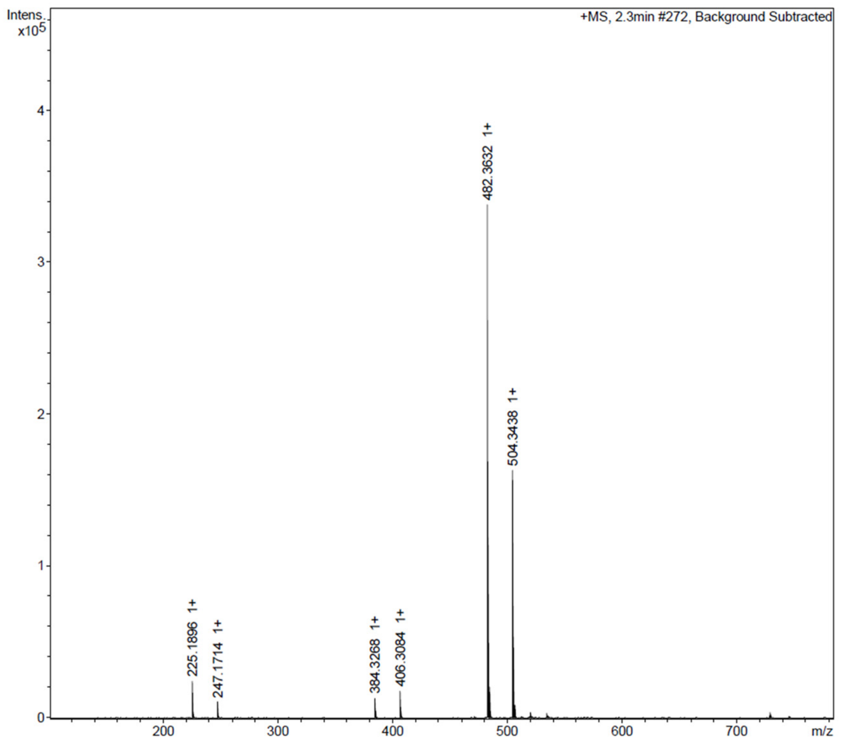

ESI-MS of DMHCA-Alk.

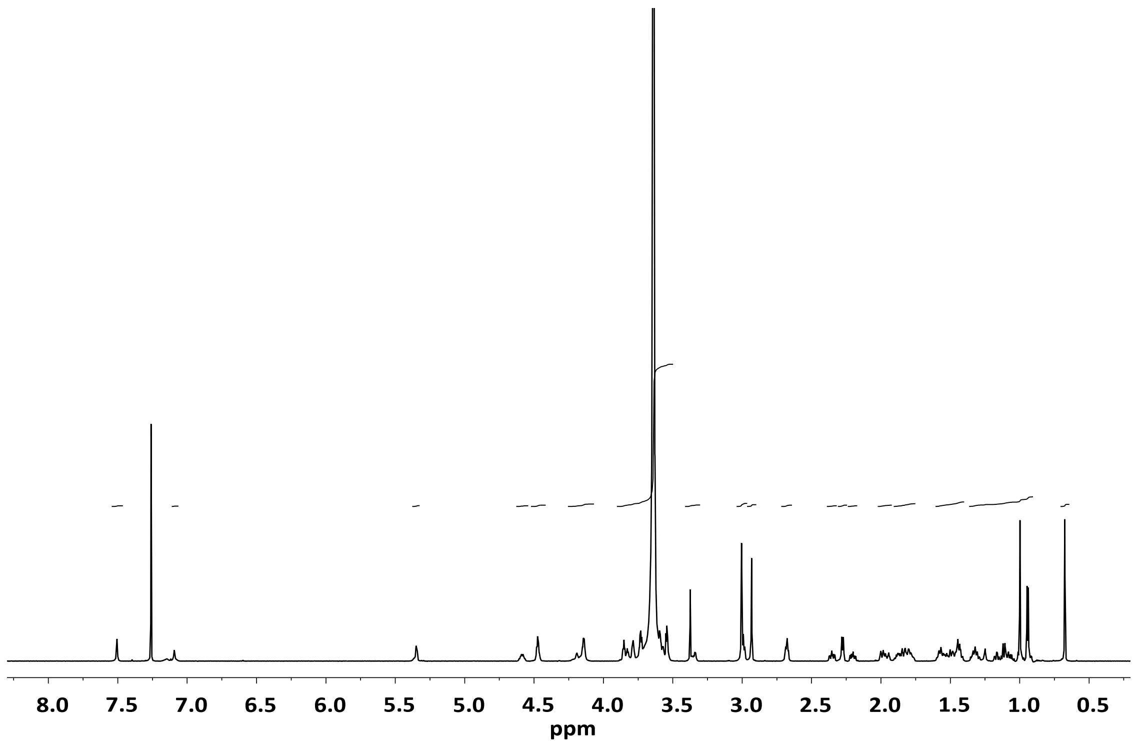

^{1}H NMR spectrum ($CDCl_3$, 750 MHz) of PEG-[G1]-DMHCA.

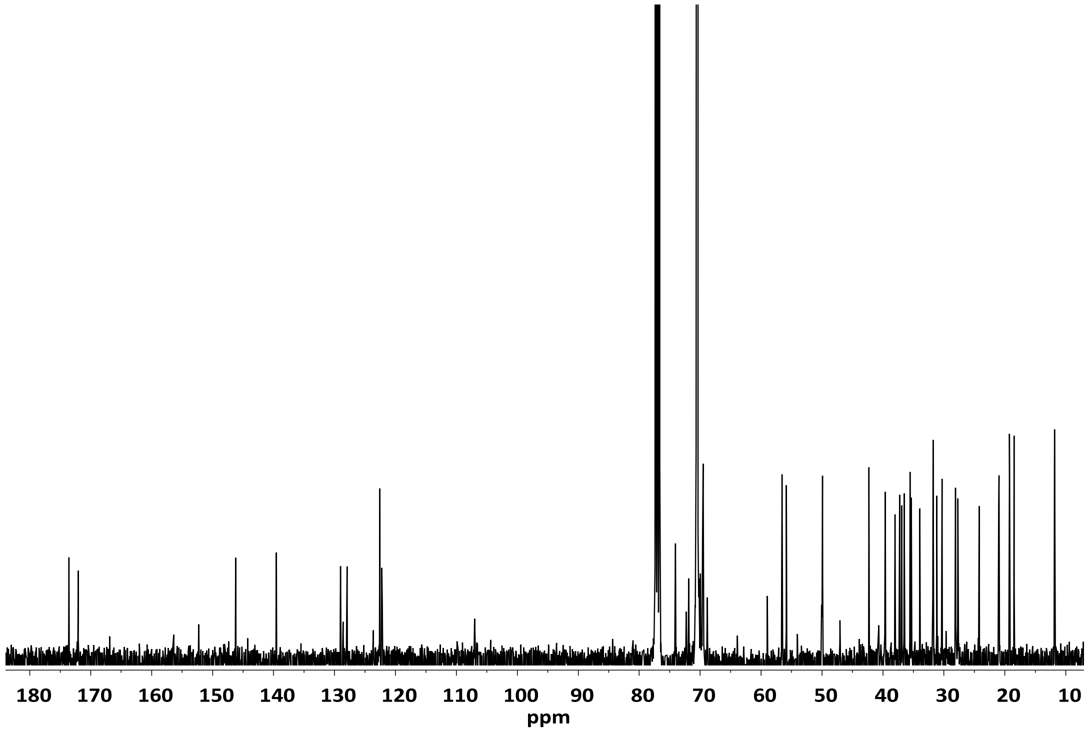

^{13}C NMR spectrum ($CDCl_3$, 100 MHz) of PEG-[G1]-DMHCA.

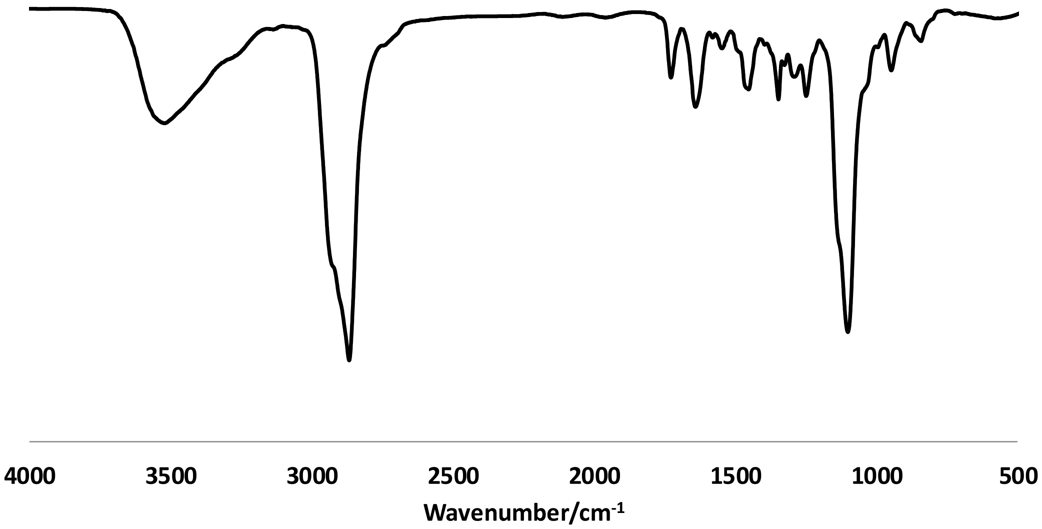

IR spectrum of PEG-[G1]-DMHCA.

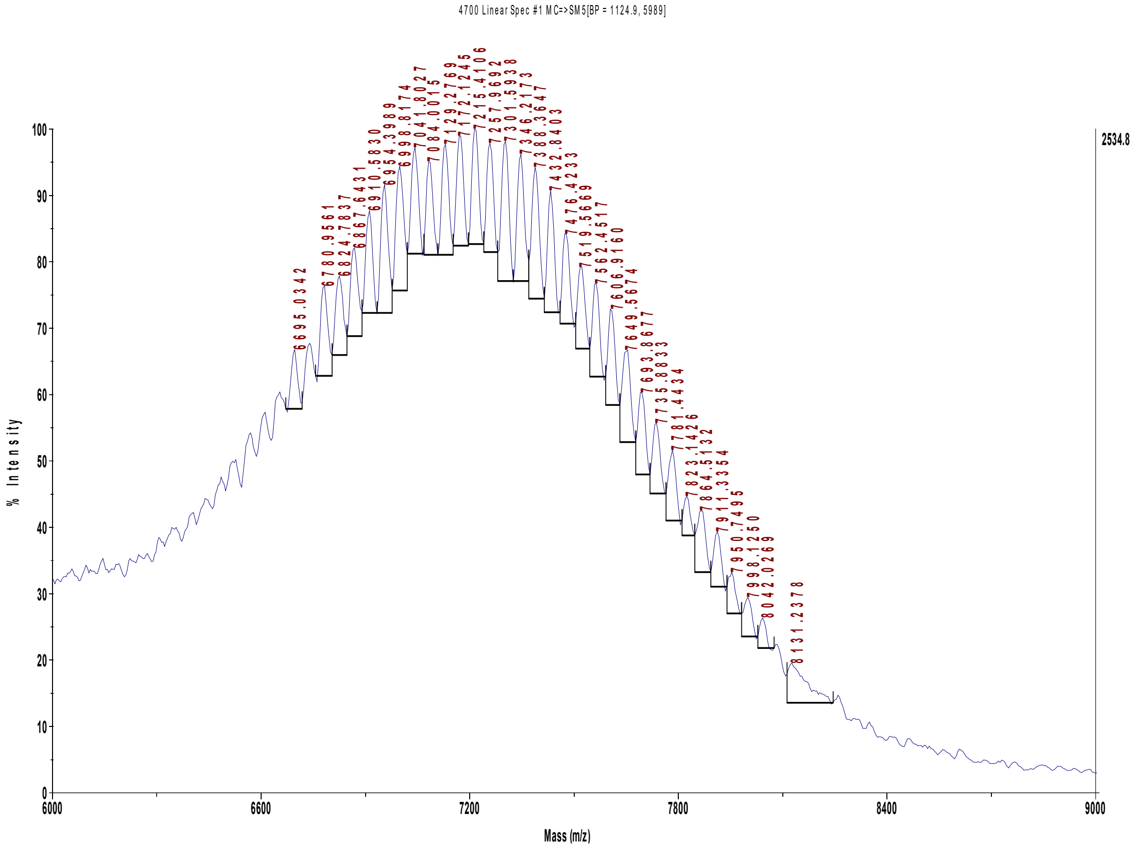

MALDI-TOF MS of PEG-[G1]-DMHCA.

6. Synthesis and Characterization of Biotin-PEG-[G1]-DMHCA

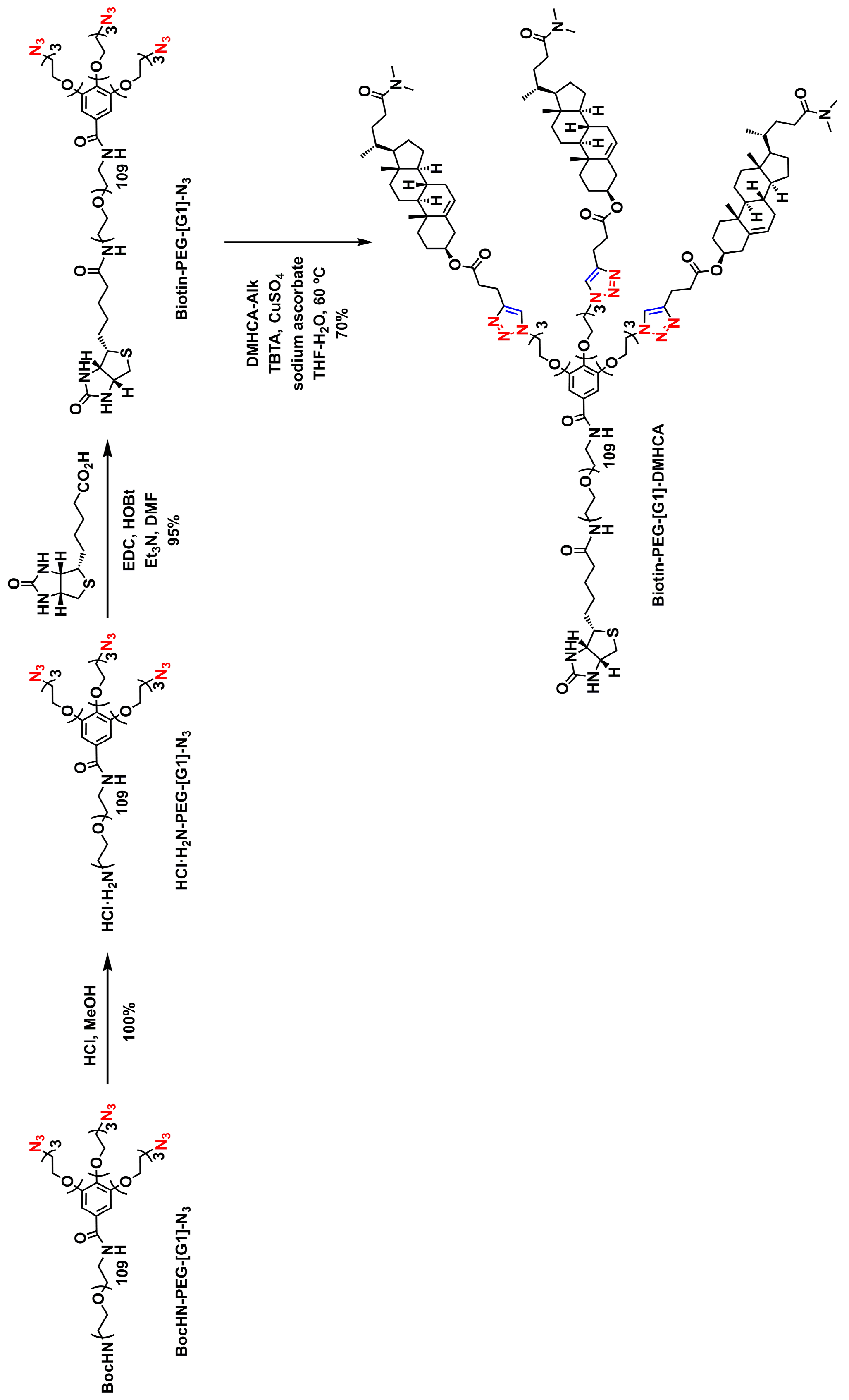

Scheme S1. Synthesis of Biotin-PEG-[G1]-DMHCA

HCl·H_2N-PEG-[G1]-N_3. BocHN-PEG-[G1]-N_3 (98 mg, 18 μmol) was dissolved in a mixture of MeOH (0.75 mL) and HCl (0.25 mL, 2.53 mmol, 37%). After 90 min of stirring at room temperature, the reaction mixture was evaporated to afford HCl·H_2N-PEG-[G1]-N_3 (99 mg, 100%) as a white solid. ^{1}H NMR (500 MHz, $CDCl_3$) δ: 7.85 (br s, 2H), 7.08 (s, 2H), 6.78 (br s, 1H), 4.25-4.17 (m, 6H), 3.92-3.55 (m, ~462H), 3.50 (t, *J*=4.8 Hz, 2H), 3.42-3.34 (m, 6H), 3.19 (br s, 2H).

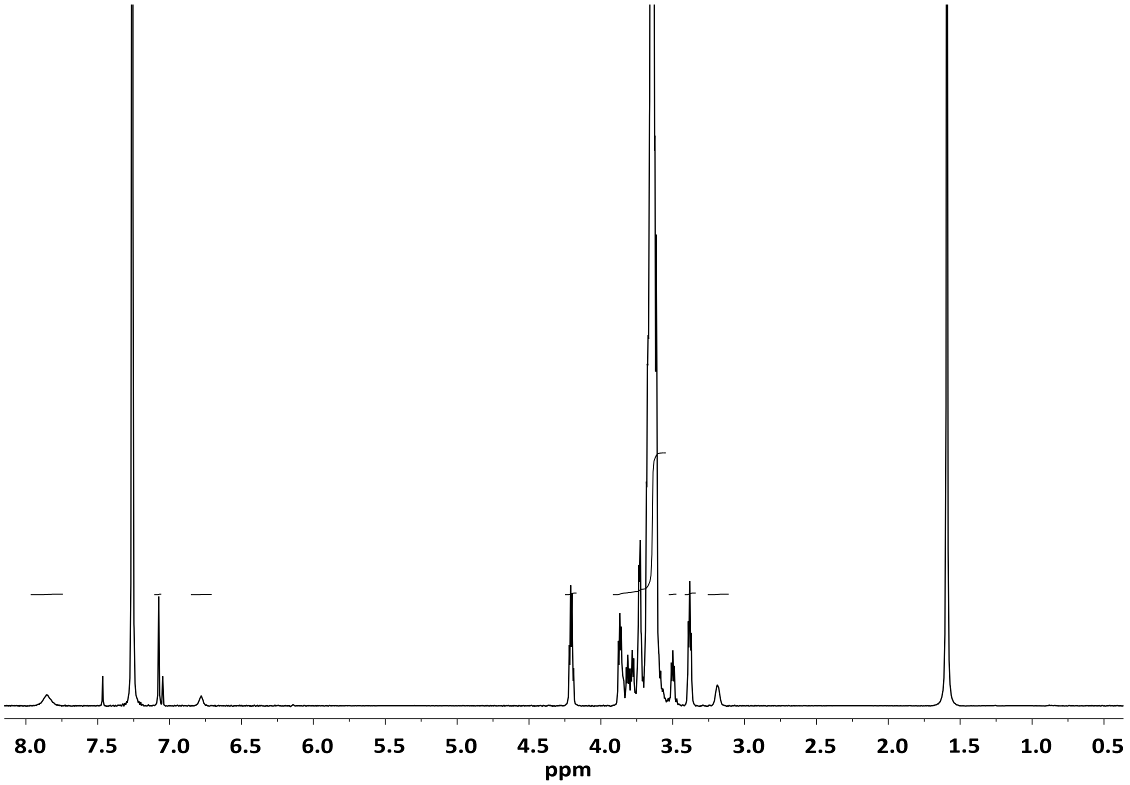

^{1}H NMR spectrum ($CDCl_3$, 500 MHz) of HCl·H_2N-PEG-[G1]-N_3.

Biotin-PEG-[G1]-N_3. Biotin (3.2 mg, 13.10 µmol), HOBt (1.8 mg, 13.10 µmol) and EDC·HCl (2.6 mg, 13.10 µmol) were added to a solution of HCl·H_2N-PEG-[G1]-N_3 (30.0 mg, 4.36 µmol) and Et_3N (4 µL, 26.19 µmol) in DMF (2.2 mL) under Ar. After 16 h of stirring at room temperature, the reaction mixture was purified by ultrafiltration (YM3) washing with H_2O (10 mL), 0.1 M $NaHCO_3$ (2 x 10 mL) and H_2O (4 x 10 mL) to give Biotin-PEG-[G1]-N_3 as a white solid after freeze-drying (29.4 mg, 95%). ^{1}H NMR (500 MHz, $CDCl_3$) δ: 7.08 (s, 2H), 6.79 (br s, 1H), 4.54-4.48 (m, 1H), 4.35-4.30 (m, 1H), 4.24-4.17 (m, 6H), 3.90-3.43 (m, ~462H), 3.41-3.34 (m, 6H), 3.21-3.14 (m, 1H), 2.93 (dd, *J*=12.7, 5.0 Hz, 1H), 2.72 (d, *J*=13.4 Hz, 1H), 2.26-2.19 (m, 2H), 1.78-1.63 (m, 1H).

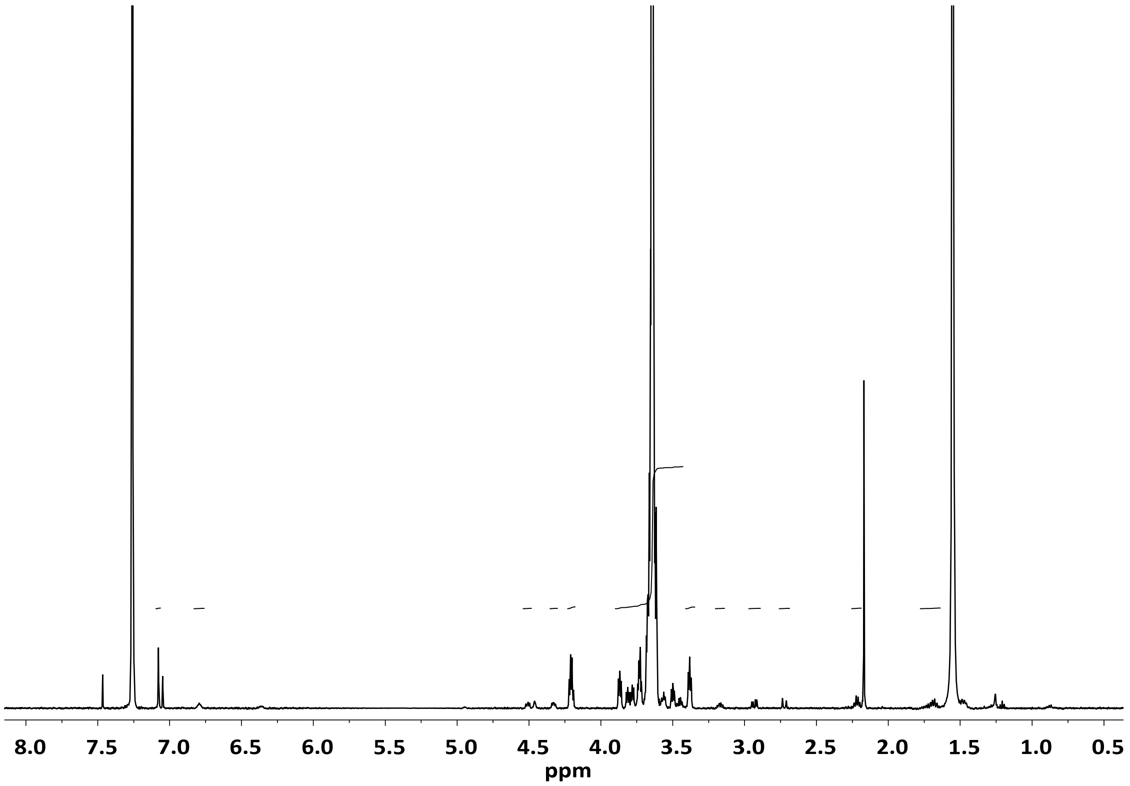

^{1}H NMR spectrum ($CDCl_3$, 500 MHz) of Biotin-PEG-[G1]-N_3.

Biotin-PEG-[G1]-DMHCA. DMHCA-Alk (16.4 mg, 34.05 μmol) was added to a solution of Biotin-PEG-[G1]-N_3 (20.9 mg, 3.78 μmol) in a mixture of THF (90 μL) and H_2O (4.2 μL). Then, TBTA (0.6 mg, 1.13 μmol), $CuSO_4$ (2.8 μL, 0.57 μmol, 0.2 M, 5 mol% per azide), and sodium ascorbate (15.0 μL, 2.83 μmol, 0.2 M, 25 mol% per azide) were added. After 12 h of stirring at 60 ºC, a second portion of sodium ascorbate (15.0 μL, 2.83 μmol, 0.2 M, 25 mol% per azide) and *t*-BuOH (20 μL) were added. After additional 24 h of stirring at 60 ºC, the reaction mixture was purified by ultrafiltration (YM3) washing with acetone:H_2O (1:1, 10 mL), acetone:0.1 M EDTA pH 7 (1:1, 5 x 10 mL), and acetone:H_2O (1:1, 5 x 10 mL) to give Biotin-PEG-[G1]-DMHCA as a white solid after freeze-drying (17.8 mg, 70%). ^{1}H NMR (500 MHz, $CDCl_3$) δ: 7.49 (s, 3H), 7.19 (br s, 1H), 7.10 (s, 2H), 6.78 (br s, 1H), 6.01 (br s, 1H), 5.34 (s, 3H), 5.06 (br s, 1H), 4.80-4.69 (m, 1H), 4.64-4.53 (m, 3H), 4.52-4.41 (m, 7H), 4.22-4.08 (m, 6H), 3.89-3.41 (m, ~462H), 3.28 (dd, *J*=13.4, 3.2 Hz, 1H), 3.08 (dd, *J*=6.1, 3.5 Hz, 2H), 3.03-2.95 (m, 16H), 2.93 (s, 9H), 2.67 (t, *J*=7.5 Hz, 6H), 2.41-2.13 (m, 14H), 2.08-0.84 (m, 90H), 0.67 (s, 9H). IR (ATR): 3437, 2867, 1727, 1642, 1639, 1103 cm^{-1}.

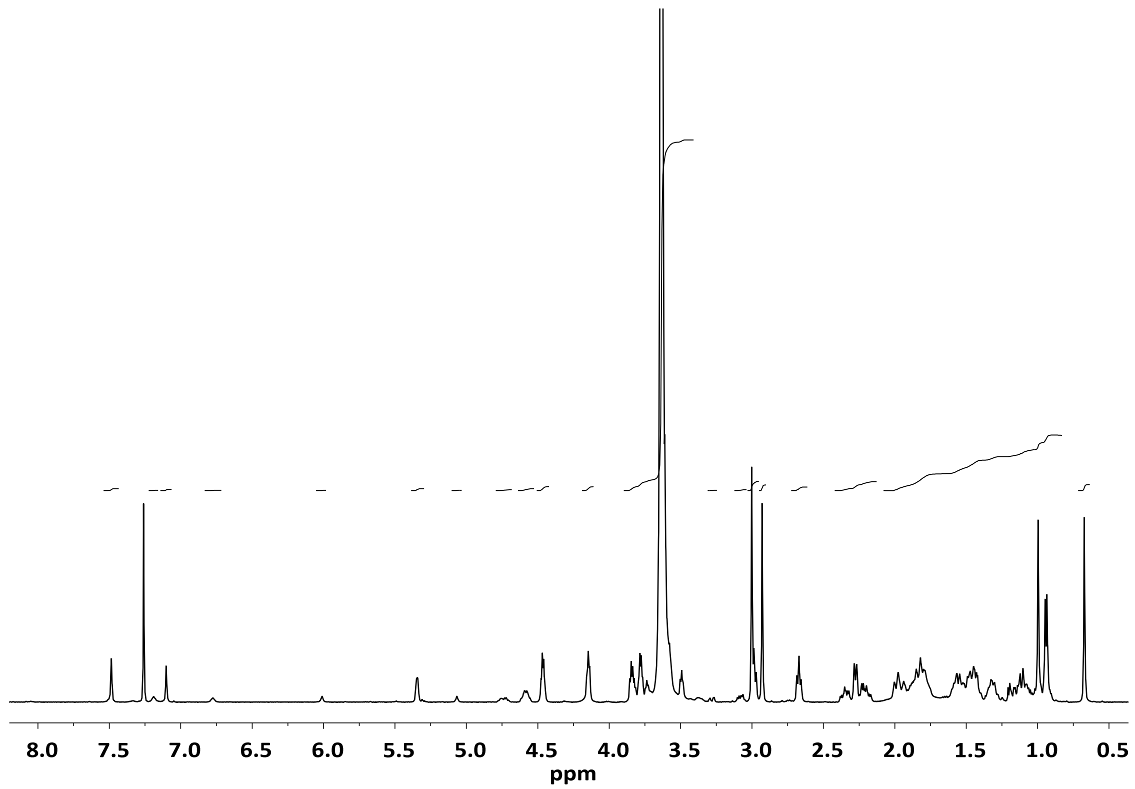

^{1}H NMR spectrum ($CDCl_3$, 500 MHz) of Biotin-PEG-[G1]-DMHCA.

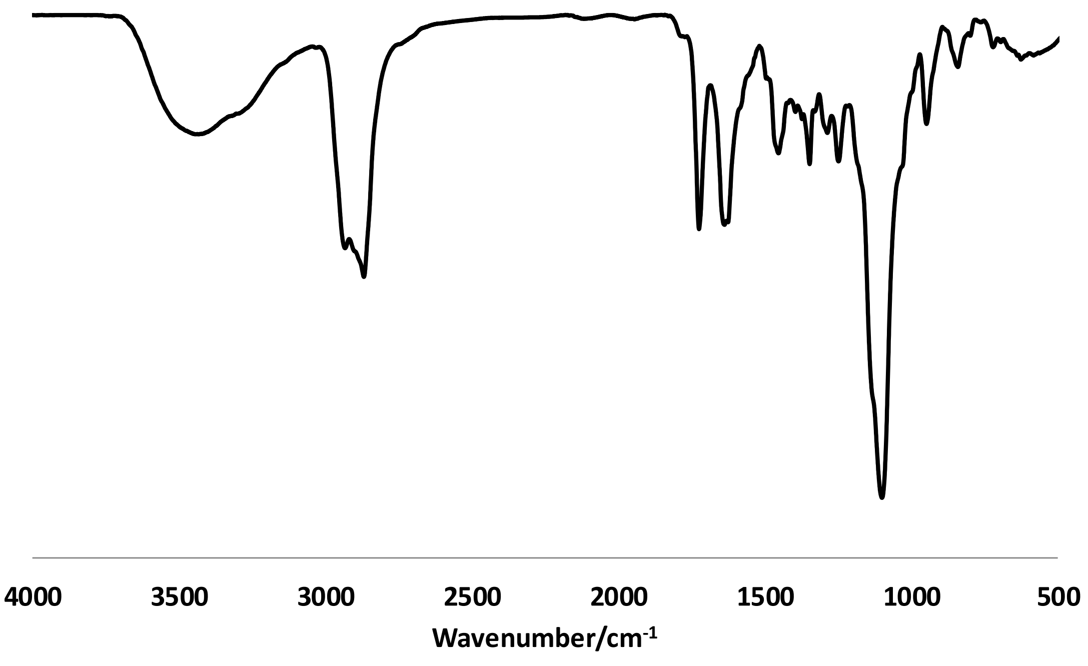

IR spectrum of Biotin-PEG-[G1]-DMHCA.

7. Assignment of Signals in IR Spectra

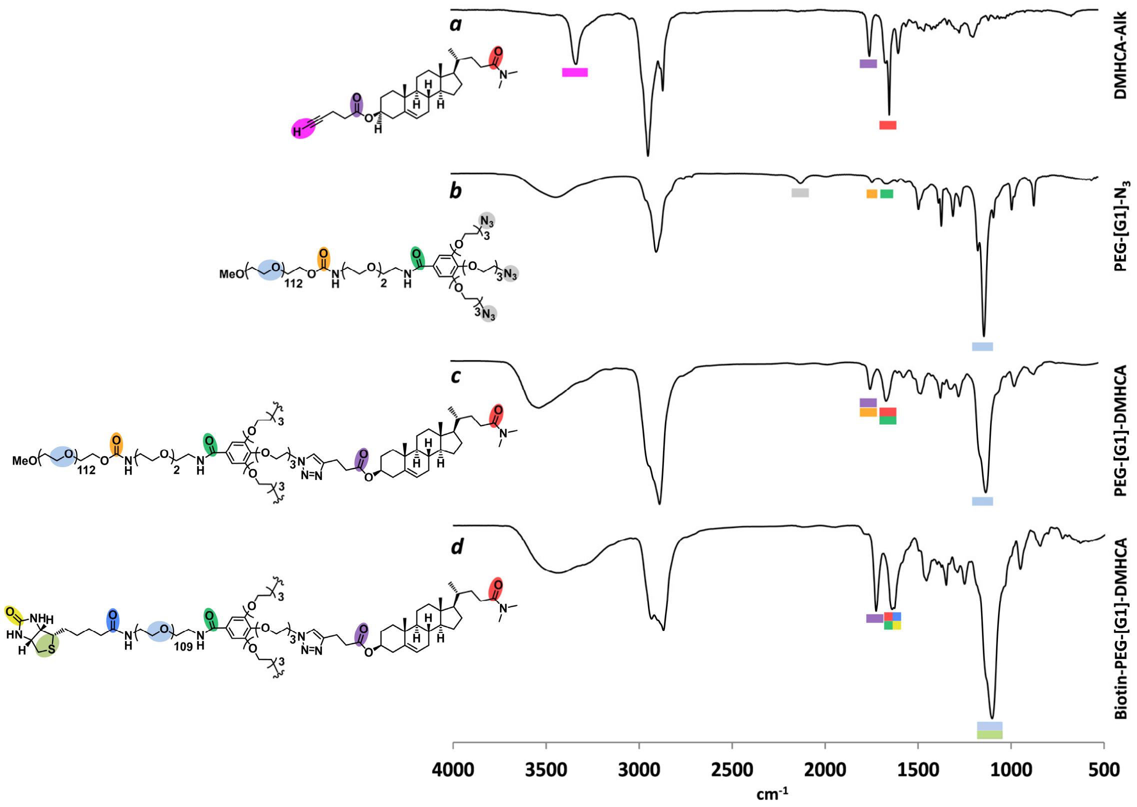

Figure S1. IR spectra of DMHCA-Alk (a), PEG-[G1]-N_3 (b), PEG-[G1]-DMHCA (c) and Biotin-PEG-[G1]-DMHCA (d).

8. DLS of PEG-[G1]-DMHCA and Biotin-PEG-[G1]-DMHCA Micelles

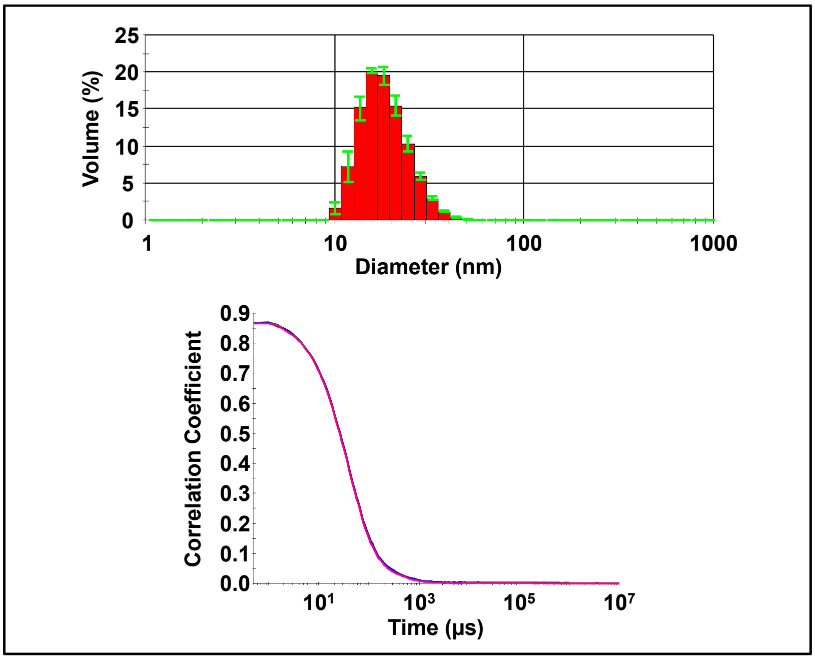

Figure S2. DLS histogram and correlation function of PEG-[G1]-DMHCA micelles upon formation (H_2O, 25 °C).

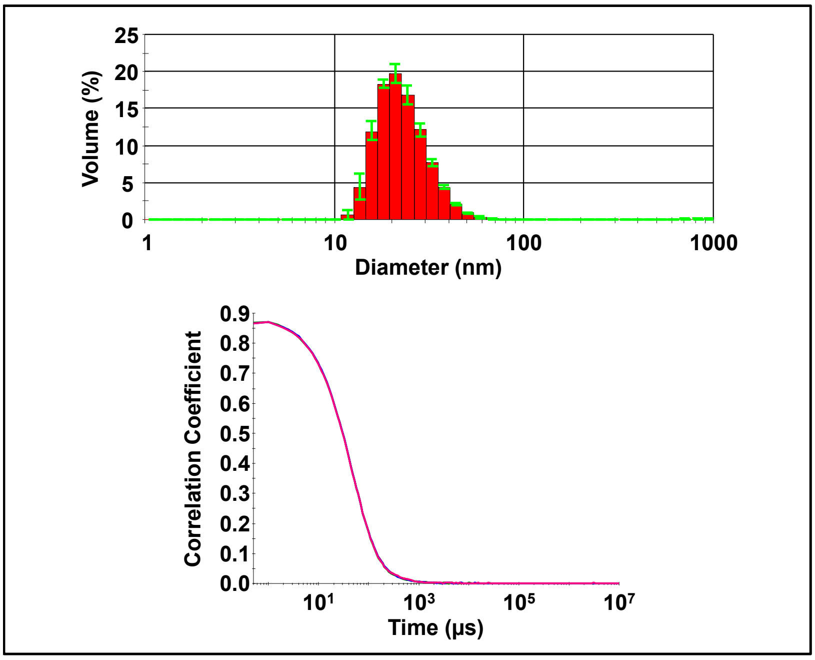

Figure S3. DLS histogram and correlation function of PEG-[G1]-DMHCA micelles after freeze-drying and resuspension (10 mM PB, pH 7.4, 150 mM NaCl; 25 °C).

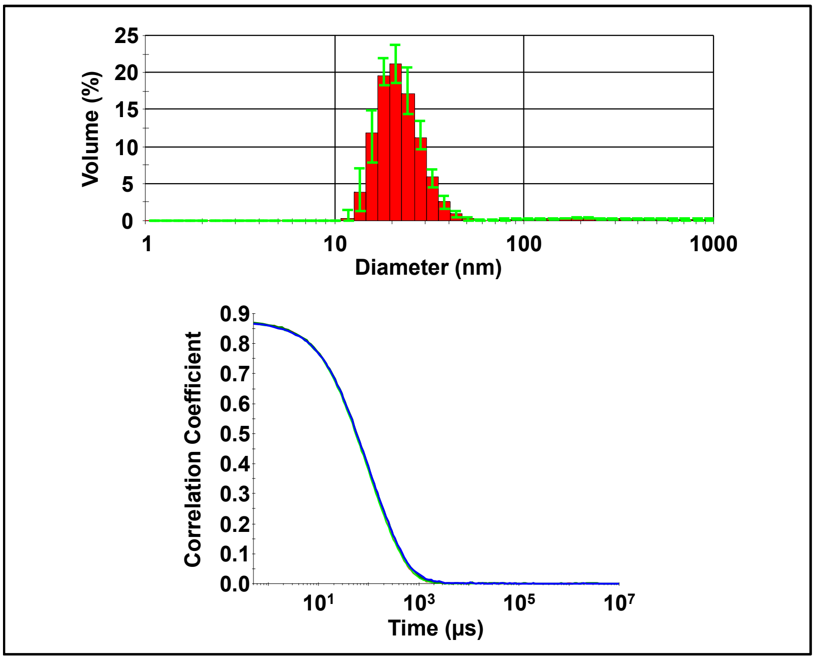

Figure S4. DLS histogram and correlation function of Biotin-PEG-[G1]-DMHCA micelles upon formation (H_2O, 25 ºC).

9. ^{1}H NMR Spectrum of PEG-[G1]-DMHCA Micelles

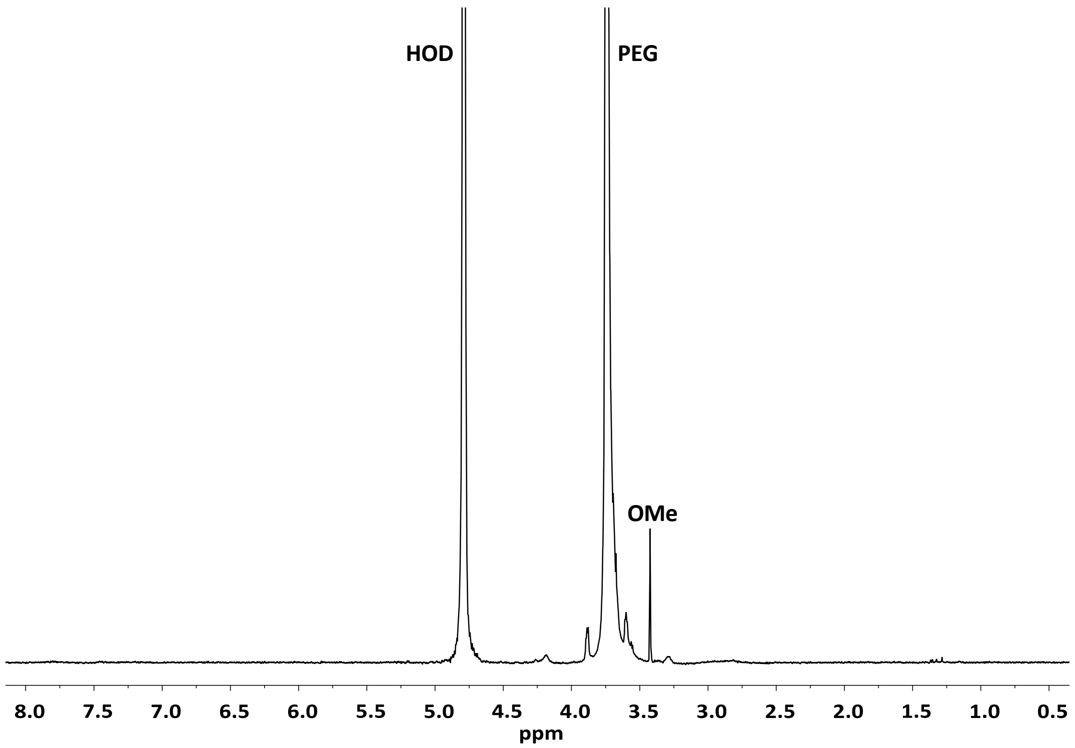

Figure S5. ^{1}H NMR spectrum (D_2O, 500 MHz, 1 mg/mL) of PEG-[G1]-DMHCA micelles after freeze-drying and resuspension in D_2O.

10. Integrity of the Ester Bond in PEG-[G1]-DMHCA Micelles by ^{1}H NMR

The NMR chemical shift of ^{1}H in alpha to oxygen atoms is very sensitive to the chemical environment. This has been exploited to study the integrity of the ester bond linking DMHCA to the dendritic block inside the micelles during their preparation, storage and lyophilization.

The ^{1}H in alpha to the hydroxyl group in DMHCA appears as a multiplet at 3.57-3.42 ppm in $CDCl_3$ (highlighted in green in Figure S6). However, after ester formation, this signal shifts to lower field: 4.71-4.56 ppm for DMHCA-Alk and 4.63-4.54 ppm for PEG-[G1]-DMHCA (highlighted in blue in Figure S6). The fact that micelles, that have been in aqueous solution for several days after preparation and then lyophilized (to remove H_2O), do not show any signal at 3.57-3.42 ppm when dissolved in $CDCl_3$, confirms the absence of free (hydrolyzed) DMHCA and so, the integrity of the ester bond during the preparation-lyophilization process.

Note that micelles disassemble to PEG-[G1]-DMHCA copolymers when dissolved in $CDCl_3$.

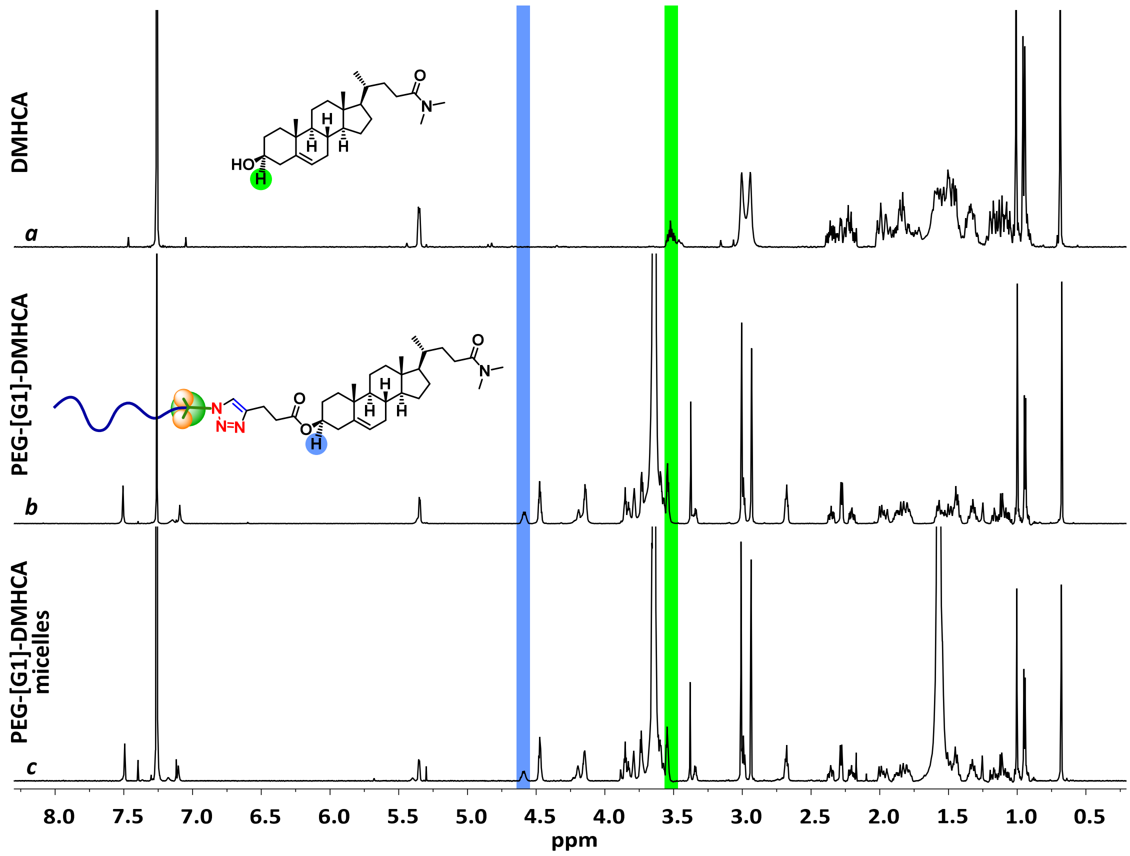

Figure S6. ^{1}H NMR spectra ($CDCl_3$) of DMHCA (500 MHz, a) and PEG-[G1]-DMHCA copolymer (750 MHz, b). ^{1}H NMR spectrum of PEG-[G1]-DMHCA micelles after freeze-drying and dissolving in $CDCl_3$ (750 MHz, c).

11. Western Blotting

Samples from the left hemisphere of wild type mice at 3 months of age were sonicated in 250 µL of lysis buffer (50 mM Tris-HCl, 150 mM NaCl, 1% Nonidet P-40, 0.1% SDS, 0.1% deoxycholic acid, 2 µg/mL of aprotinin, 2 µg/mL of leupeptin, 100 µg/mL PMSF, pH 7.4). Samples were centrifuged at 13000 rpm for 45 min at 4 ºC and protein content of the supernatants was determined using the Dc-protein assay, (BioRad, USA). 50-150 µg of total protein were loaded on a 10-20% acrylamide gel and transferred to a PVDF membrane. The membranes were blocked for 1 h at room temperature in 5% skim milk and then incubated overnight at 4 ºC with the primary antibody (rabbit polyclonal anti-ApoE, working dilution 1:1000, Abcam; rabbit polyclonal anti-ABCA1, working dilution 1:500, Abcam; mouse monoclonal 6E10, working dilution 1:1000, Millipore). After washing in TBS-T buffer, the membranes were incubated with goat anti-rabbit or anti-mouse peroxidase-conjugated secondary antibody (working dilution 1:5000, BioRad, USA) for 1 h at room temperature. βIII-tubulin (working dilution: 1:12000, Abcam) or GAPDH (glyceraldehyde-3-phosphate dehydrogenase, working dilution: 1:5000, Abcam) were used as loading controls. Signal intensity was quantified by densitometry (Image Studio Lite). Groups were obtained simultaneously and normalized to βIII-tubulin (neuronal specific) immunoreactivity. All experiments were performed in triplicate.

12. Immunohistochemistry

Free floating immunohistochemical staining was performed as previously described.[7] Mouse monoclonal primary antibodies used were: McSA1 (from MediMabs, Montreal, Canada, working dilution: 1:1500), which recognizes the first 12 amino acid of the amyloid beta sequence. For mouse monoclonal primary antibodies, sections were

incubated with a secondary goat-anti-mouse IgG (working dilution 1:1000; BioRad, USA). Staining were then developed with 0.06% DAB (Cell Marque, USA).

Digital images were acquired on Imaging microscope (Olympus), equipped with a Camera HRc (Olympus), using Image Pro Plus software for quantification (MediaCybernetics).

13. *In Vivo* Cytotoxicity

The potential *in vivo* cytotoxicity of the intranasal experimental doses of PEG-[G1]-DMHCA micelles was evaluated in mice at different time-points (Figure S7) and in 21 days treated transgenic mice (Figure S8) using a colorimetric caspase 3/7 cytotoxicity assay kit (Abcam, ab39401). This assay is based on the formation of the chromophore p-nitroaniline (p-NA) by cleavage from the labeled substrate DEVD-pNA. The p-NA can be quantified using a spectrophotometer, reading the absorbance at 405 nm. Homogenates from different tissues were processed following the manufacturer recommendation. Briefly, homogenates (50 μg/well), reaction buffer and DEVD-p-NA substrate were added to the wells and incubated for 90 min at 37 ºC. Plates were analyzed in a microplate reader at 405 nm. Each sample was analyzed by triplicate.

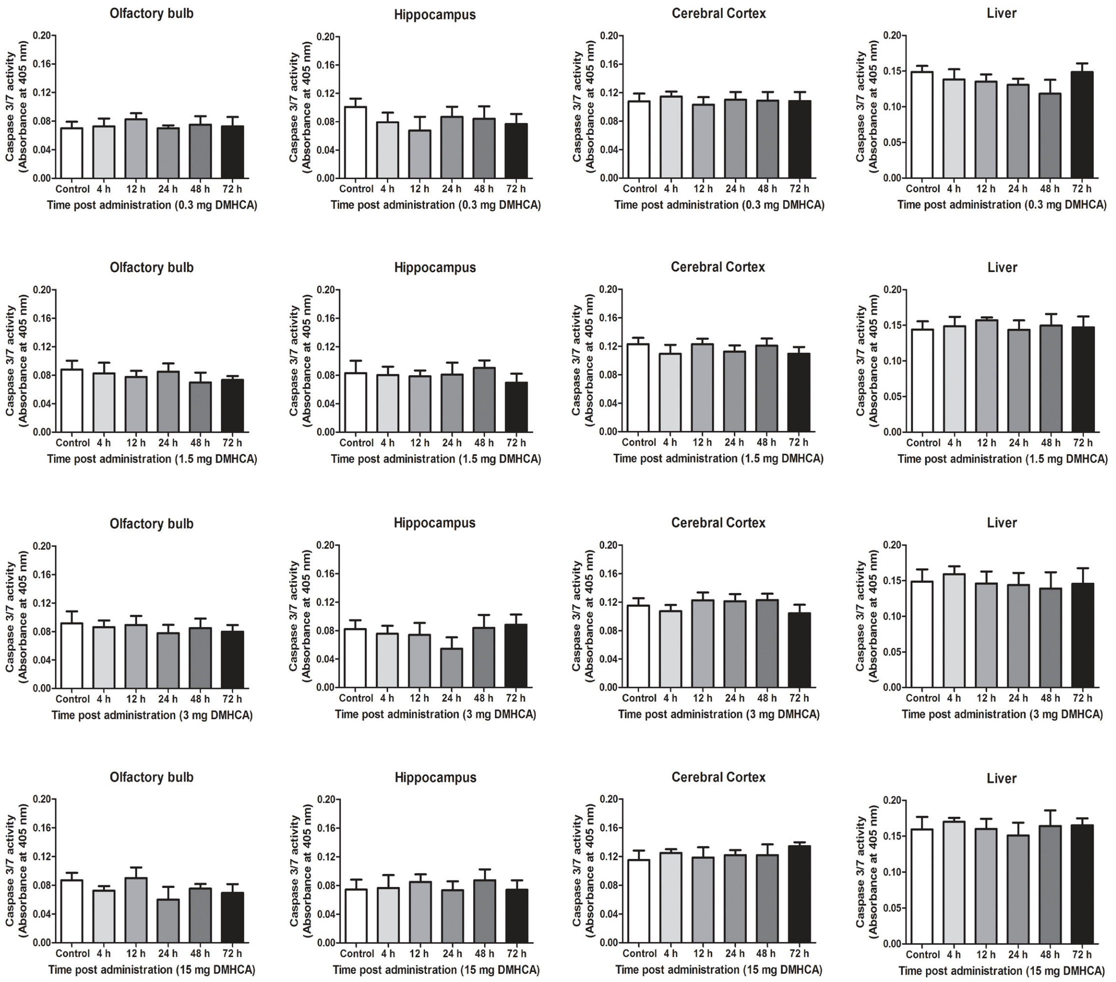

Figure S7. No *in vivo* cell toxicity was shown at different time-points (4, 12, 24, 48 and 72 h) by caspase 3/7 activity after intranasal dose administration in mice (0.3, 1.5, 3 and 15 mg DMHCA/kg body weight/day, equivalent to 1.5, 7.5, 15 and 75 mg of PEG-[G1]-DMHCA/kg body weight/day). Relative caspase 3/7 activity in homogenates of olfactory bulb, hippocampus, cerebral cortex and liver. Statics analysis were performed using Graph-Pad Prism 6. All probability values were two-tailed; a level of 5% was considered significant. Data are reported as the mean ± SEM.

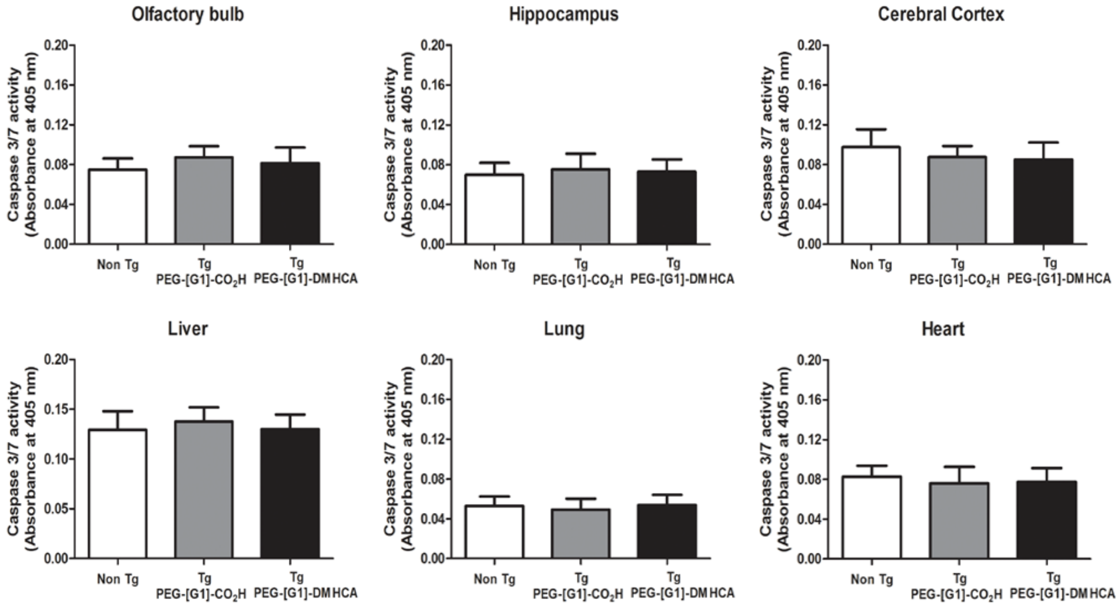

Figure S8. Absence of cytotoxicity induced by PEG-[G1]-DMHCA micelles after long-term (21 days) intranasal treatment. Homogenates (brain, liver, lungs and heart) from experimental non-transgenic (non Tg), and transgenic (Tg) mice treated with PEG-[G1]-CO_2H (control) and PEG-[G1]-DMHCA (experimental) were analyzed. Not significant statistical differences in caspase 3/7 activity were observed among tissues from the three experiment groups. Statics analysis were performed using Graph-Pad Prism 6. All probability values were two-tailed; a level of 5% was considered significant. Data are reported as the mean ± SEM.

14. Data Analysis

All data were analyzed using the Graph-Pad Prism 6 software. Comparison was done by unpaired Student's T-test. Significance was set at $p<0.05$. Data are presented as mean ± S.E.M.

15. References

1. Fernandez-Megia, E.; Correa, J.; Riguera, R. "Clickable" PEG-Dendritic Block Copolymers. *Biomacromolecules* **2006**, *7*, 3104-3111.

2. Fernandez-Villamarin, M.; Sousa-Herves, A.; Correa, J.; Munoz, E. M.; Taboada, P.; Riguera, R.; Fernandez-Megia, E. The Effect of PEGylation on Multivalent Binding: A Surface Plasmon Resonance and Isothermal Titration Calorimetry Study with Structurally Diverse PEG-Dendritic GATG Copolymers. *ChemNanoMat* **2016**, *2*, 437-446.

3. Fernandez-Villamarin, M.; Sousa-Herves, A.; Porto, S.; Guldris, N.; Martínez-Costas, J.; Riguera, R.; Fernandez-Megia, E. A Dendrimer–Hydrophobic Interaction Synergy Improves the Stability of Polyion Complex Micelles. *Polym. Chem.* **2017**, *8*, 2528-2537.

4. Sousa-Herves, A.; Sanchez Espinel, C.; Fahmi, A.; Gonzalez-Fernandez, A.; Fernandez-Megia, E. *In Situ* Nanofabrication of Hybrid PEG-Dendritic-Inorganic Nanoparticles and Preliminary Evaluation of Their Biocompatibility. *Nanoscale* **2015**, *7*, 3933-3940.

5. Sousa-Herves, A.; Fernandez-Megia, E.; Riguera, R. Synthesis and Supramolecular Assembly of Clicked Anionic Dendritic Polymers into Polyion Complex Micelles. *Chem. Commun.* **2008**, *27*, 3136-3138.

6. Karaki, F.; Ohgane, K.; Dodo, K.; Hashimoto, Y. Structure-Activity Relationship Studies of Niemann-Pick Type C1-Like 1 (NPC1L1) Ligands Identified by Screening Assay Monitoring Pharmacological Chaperone Effect. *Bioorg. Med. Chem.* **2013**, *21*, 5297-5309.

7. Hu, L.; Wong, T. P.; Cote, S. L.; Bell, K. F.; Cuello, A. C. The Impact of Abeta-Plaques on Cortical Cholinergic and Non-Cholinergic Presynaptic Boutons in Alzheimer's Disease-Like Transgenic Mice. *Neuroscience* **2003**, *121*, 421-432.
